# Network Pharmacology, Molecular Dynamics Simulation, and Biological Validation Insights into the Potential of Ligustri Lucidi Fructus for Diabetic Nephropathy

**DOI:** 10.3390/ijms26136303

**Published:** 2025-06-30

**Authors:** Manting Liu, Yuhao Gu, Yuchang Yang, Ke Zhang, Jingwen Yang, Wenqi Wang, Wenjing Li, Xinzhu Wang, Xiaoxv Dong, Xingbin Yin, Changhai Qu, Boran Ni, Jian Ni

**Affiliations:** 1 School of Chinese Materia Medica, Beijing University of Chinese Medicine, Beijing 102401, China; 20230941503@bucm.edu.cn (M.L.); 20220941456@bucm.edu.cn (Y.G.); 20240941529@bucm.edu.cn (Y.Y.); 20240935273@bucm.edu.cn (K.Z.); 20240935160@bucm.edu.cn (J.Y.); 20240935097@bucm.edu.cn (W.W.); 20240935159@bucm.edu.cn (W.L.); 20240935161@bucm.edu.cn (X.W.); 201801020@bucm.edu.cn (X.D.); yxbtcm@bucm.edu.cn (X.Y.); quchanghai@bucm.edu.cn (C.Q.); 2 China Academy of Chinese Medical Sciences, Beijing 100700, China

**Keywords:** Ligustri Lucidi Fructus, diabetic nephropathy, network pharmacology, molecular docking, molecular dynamics simulation, cGAS-STING

## Abstract

Diabetic nephropathy (DN) represents a severe microvascular complication of diabetes mellitus. As a Traditional Chinese Medicine (TCM) with extensive clinical applications, Ligustri Lucidi Fructus (LLF) exhibits significant anti-DN activity. However, the underlying pharmacological mechanisms, crucial components, and targets for LLF in DN treatment remain unclear. By integrating network pharmacology, molecular docking, and molecular dynamics simulations, the bioactive compounds, potential therapeutic targets, and underlying mechanisms of LLF in the treatment of DN were elucidated, followed by biological validation in a palmitic acid (PA)-induced MPC5 podocyte injury model. Among the 383 DN-related LLF targets identified, TNF emerged as a pivotal one, demonstrating potential binding interaction with the active components salidroside (Sal), apigenin (Api), and tormentic acid (TA). Moreover, Gene Expression Omnibus (GEO) database and KEGG enrichment analysis collectively highlighted the cytosolic DNA-sensing pathway. Notably, the cGAS-STING pathway is central to this pathway. Experimental studies further demonstrated that LLF-containing serum exerted a protective effect on MPC5 podocytes through cGAS-STING pathway suppression. Overall, these findings elucidate the pleiotropic mechanisms underlying LLF’s protective effects against DN, integrating compound–target–pathway interactions and thus offering a rationale for further investigation.

## 1. Introduction

As a severe microvascular complication of diabetes, diabetic nephropathy (DN) is the primary cause of end-stage kidney disease (ESKD) and chronic kidney disease (CKD) [1]. Epidemiological studies indicate that the global incidence of DN varies between 23.4% and 47.11% [2]. The pathogenesis of DN includes podocyte injury, metabolic and hemodynamic dysregulation, inflammation driven by reactive oxygen species (ROS), and the accumulation of Advanced Glycation End-products (AGEs) [3,4,5]. Currently, the therapeutic options for DN remain limited [6]. In clinical settings, the management of DN involves hypoglycemic, lipid-lowering, antihypertensive, and renoprotective agents, either as monotherapies or in combination therapy. However, pharmacological interventions such as these frequently lead to adverse reactions [7,8]. Accordingly, the discovery of novel therapeutic agents with improved potency and favorable safety profiles constitutes a crucial imperative.

Traditional Chinese Medicine (TCM) has been extensively used in the management of diabetes and its complications and demonstrated remarkable therapeutic efficacy. Ligustri Lucidi Fructus (LLF), derived from the dry ripe fruit of *Ligustrum lucidum* W.T. Aiton [9], demonstrates diverse pharmacological properties including anti-tumor [10], immunomodulatory [11], anti-inflammatory [12,13], hypolipidemic [14,15], and anti-osteoporotic effects [16,17]. Experimental studies have revealed that both LLF-derived polyphenols and aqueous extract significantly reduce hyperglycemia [18,19]. Additionally, components of LLF, notably salidroside [20,21,22], oleanolic acid [23,24], and ursolic acid [25,26], potentially exert effects against DN. Despite these findings, the precise targets and underlying mechanisms of LLF’s role in DN prevention require further investigation.

**Figure 1:**
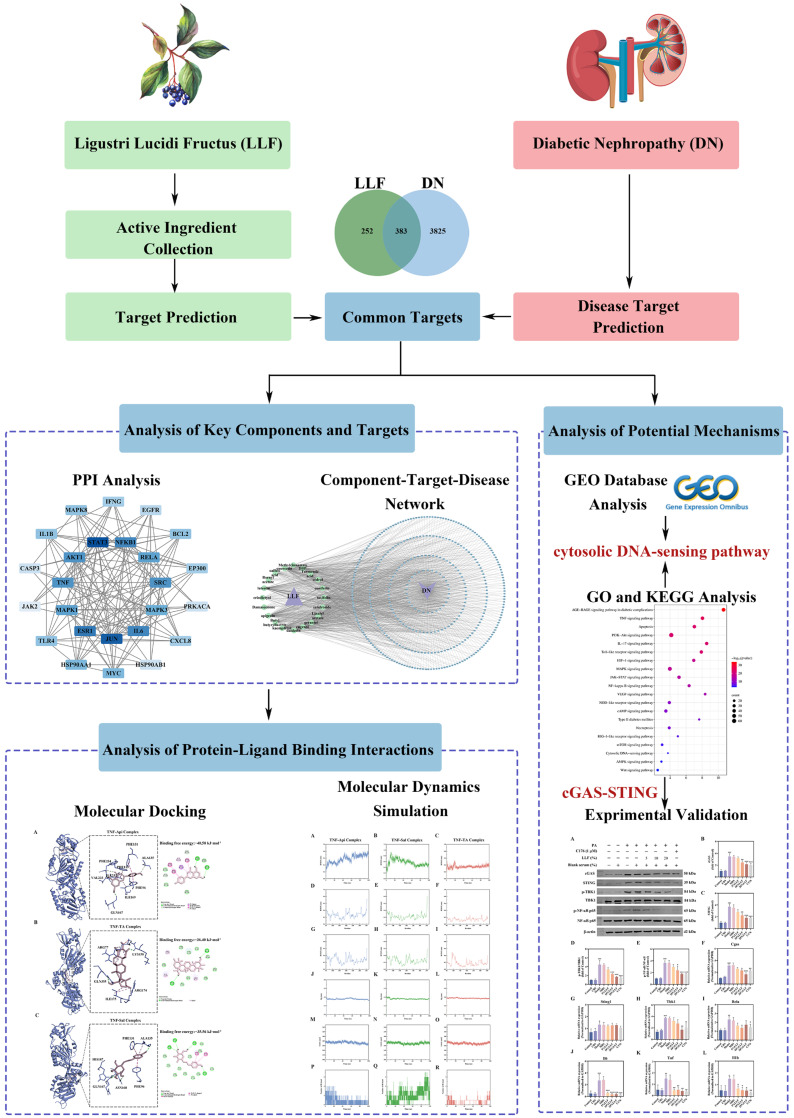
Crucial components, targets, and mechanisms of Ligustri Lucidi Fructus (LLF) in diabetic nephropathy (DN) revealed by integrated network pharmacology and experimental validation. Workflow includes (1) screening 383 DN-related targets in LLF, (2) protein–protein interaction (PPI) and component–target–disease network analyses identifying core targets and components, (3) molecular docking and dynamics simulations confirming protein–ligand interactions, (4) GEO and KEGG analyses implicating the cytosolic DNA-sensing pathway, and (5) experimental validation of the podocyte-protective effects of LLF-containing serum and the mechanisms associated with this.

Drugs such as LLF form complex pharmacological systems that exhibit intricate networks of synergistic and antagonistic interactions. Currently, computational technologies, particularly network pharmacology [27,28], molecular docking [29], and molecular dynamics simulation [30], have shown a remarkable capability for elucidating the regulatory networks of TCM. These approaches, spanning from systemic network analysis to specific target investigation and from static binding prediction to dynamic interaction profiling, progressively unravel the molecular mechanisms underlying drug actions. Therefore, integrated with network pharmacology, molecular docking, and molecular dynamics simulation, the bioactive compounds, potential therapeutic targets, and underlying signaling pathways of LLF in DN treatment were systematically clarified (Figure 1). In order to investigate the protective effects of LLF on podocytes, a palmitic acid (PA)-induced MPC5 podocyte injury model was established. Additionally, the cGAS-STING pathway was experimentally validated as being involved in the mechanism underlying LLF’s effects. Collectively, this study aimed to provide a mechanistic basis for further the investigation of LLF for DN treatment.

## 2. Results

### 2.1. Active Compounds in LLF and Their Targets

In this study, 92 compounds were retrieved from the TCMSP and HERB databases. Among them, 20 bioactive compounds were identified based on drug-likeness and pharmacokinetic criteria (Table S1). A total of 635 potential targets for these 20 active compounds were predicted through the SwissTargetPrediction, TCMSP, and HERB databases.

### 2.2. Effective Targets for DN

**Figure 2:**
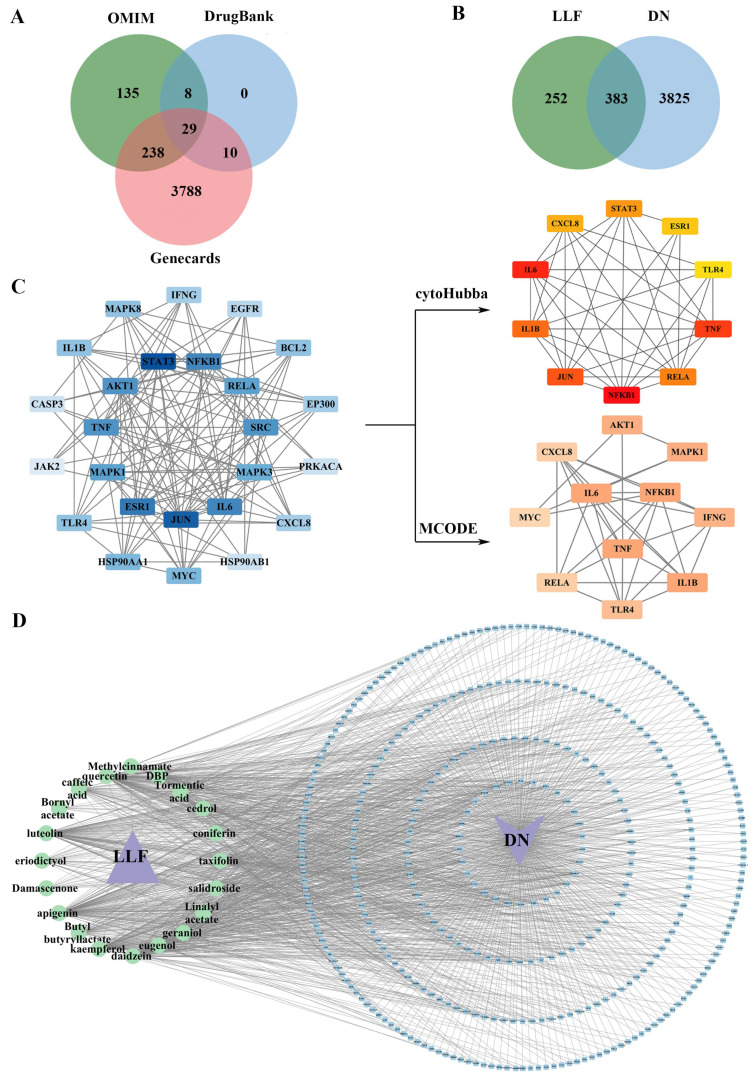
Active components of Ligustri Lucidi Fructus (LLF) and their key targets in the treatment of diabetic nephropathy (DN). (**A**) Venn diagram of DN-related targets. (**B**) Venn diagram of 383 anti-DN targets of LLF. (**C**) Protein–protein interaction (PPI) network based on MCODE and cytoHubba plug-ins. (**D**) “Component–target–disease” network. In the figure, purple triangle represents drug, purple V-shape denotes disease, green ellipses indicate molecules, and blue round rectangles correspond to genes.

A total of 4208 DN-related targets were obtained by combining data from the OMIM, DrugBank, and GeneCards databases (Figure 2A). Through intersection analysis, 383 overlapping targets were identified, implicating them in the therapeutic effects of LLF in DN (Figure 2B).

### 2.3. Establishment of the PPI Network with Common Targets

A protein–protein interaction (PPI) network of the 383 common targets was constructed containing 308 nodes and 1430 edges (Figure S1). Based on the median values of betweenness centrality, closeness centrality, and degree, 25 targets were initially identified (Figure 2C). Highly interconnected sub-networks were subsequently established via the MCODE and cytoHubba plug-ins (Figure 2C). The MCODE sub-network included IFNG, MAPK1, TNF, RELA, TLR4, AKT1, CXCL8, IL6, IL1B, and MYC, whereas the cytoHubba sub-network highlighted IL6, IL1B, JUN, STAT3, CXCL8, RELA, TLR4, TNF, NFKB1, and ESR1. The seven targets (NFKB1, TNF, TLR4, CXCL8, IL6, IL1B, and RELA) present in both sub-networks were determined as the core targets in LLF’s anti-DN effects.

### 2.4. Integrated “Component–Target–Disease” Network Analysis

A “component–target–disease” interaction network was constructed, consisting of 405 nodes and 1430 edges (Figure 2D). The top five compounds ranked by degree were quercetin (degree: 138), apigenin (degree: 129), luteolin (degree: 113), kaempferol (degree: 103), and daidzein (degree: 101) (Table S2).

### 2.5. KEGG Analysis of Common Targets

**Figure 3:**
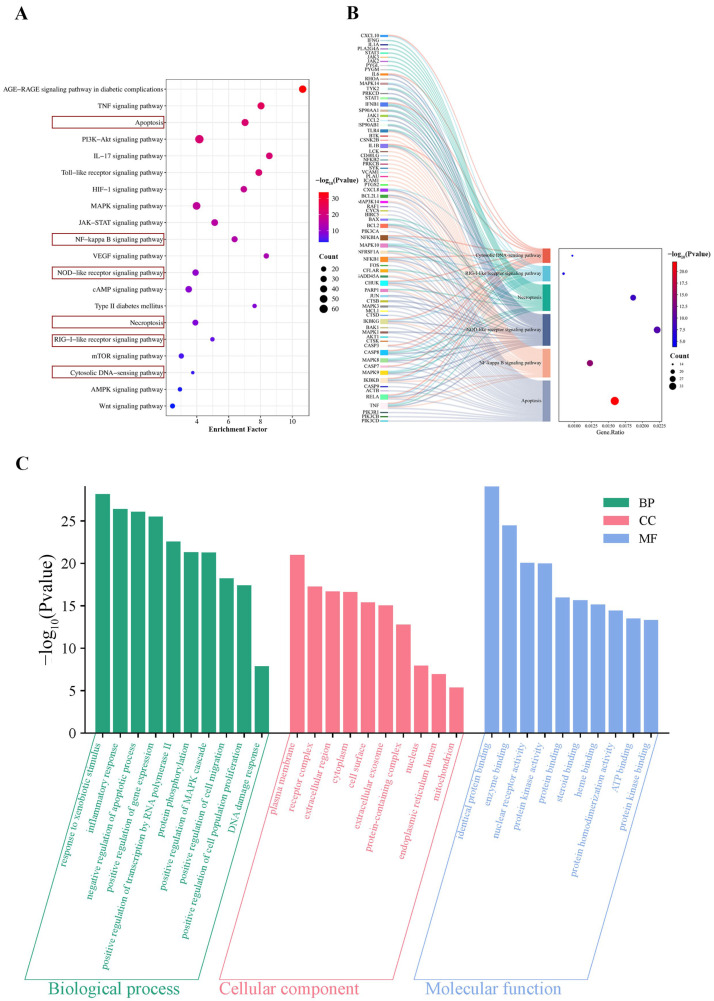
Gene Ontology (GO) and KEGG pathway analysis for 383 overlapping targets. (**A**) Top 20 significantly enriched KEGG pathways (*p*-value < 0.05). The pathways highlighted within the red box represent the six pathways displayed in the Sankey diagram. (**B**) Sankey diagram illustrating the relevant pathways in the map of the cytosolic DNA-sensing pathway (map04623). (**C**) Top 10 terms in GO enrichment analyses (*p*-value < 0.05).

A total of 186 significant pathways (*p*-value < 0.05) were identified through functional analysis of the 383 overlapping targets. The top 20 DN-associated pathways ranked by *p*-value were selected for visualization (Table S3, Figure 3A). Among them, the apoptosis, NF-κB signaling, NOD-like receptor signaling, necroptosis, and RIG-I-like receptor signaling pathways were all associated with the cytosolic DNA-sensing pathway (KEGG entry: map04623) in the KEGG database (Figure S2). A Sankey diagram was generated to illustrate the interactions between these six pathways and their associated targets (Figure 3B). These findings suggested that the cytosolic DNA-sensing pathway may be important in the therapeutic effects of LLF against DN.

### 2.6. GO Analysis of Common Targets

A total of 976 biological process (BP) terms, 116 cellular component (CC) terms, and 249 molecular function (MF) terms (*p*-value < 0.05) were identified through Gene Ontology (GO) analysis. The top 10 enriched terms were chosen for visualization (Table S4, Figure 3C). The enriched BP terms were primarily associated with responses to xenobiotic stimuli and inflammation, regulation of apoptosis and gene expression, transcription by RNA polymerase II, protein phosphorylation, MAPK cascade activation, cell migration, cell proliferation, and the DNA damage response. The top 10 CC terms mainly involved the plasma membrane, protein-containing complexes, the extracellular region, the cytoplasm, the cell surface, extracellular exosomes, the nucleus, the endoplasmic reticulum lumen, and the mitochondria. In addition, the enriched MF terms included identical protein binding, enzyme binding, nuclear receptor activity, protein kinase activity, steroid binding, protein homodimerization activity, ATP binding, and protein kinase binding.

### 2.7. GEO Analysis of the GSE142025 Dataset

Based on the GSE142025 dataset, the therapeutic potential of LLF against DN was further investigated. The dataset included 6 kidney biopsy samples from patients with early-stage DN, 21 from patients with advanced-stage DN, and 9 normal kidney samples obtained from nephrectomy specimens. Accordingly, the samples were categorized into three groups (control, early DN, and advanced DN) for subsequent analysis.

**Figure 4:**
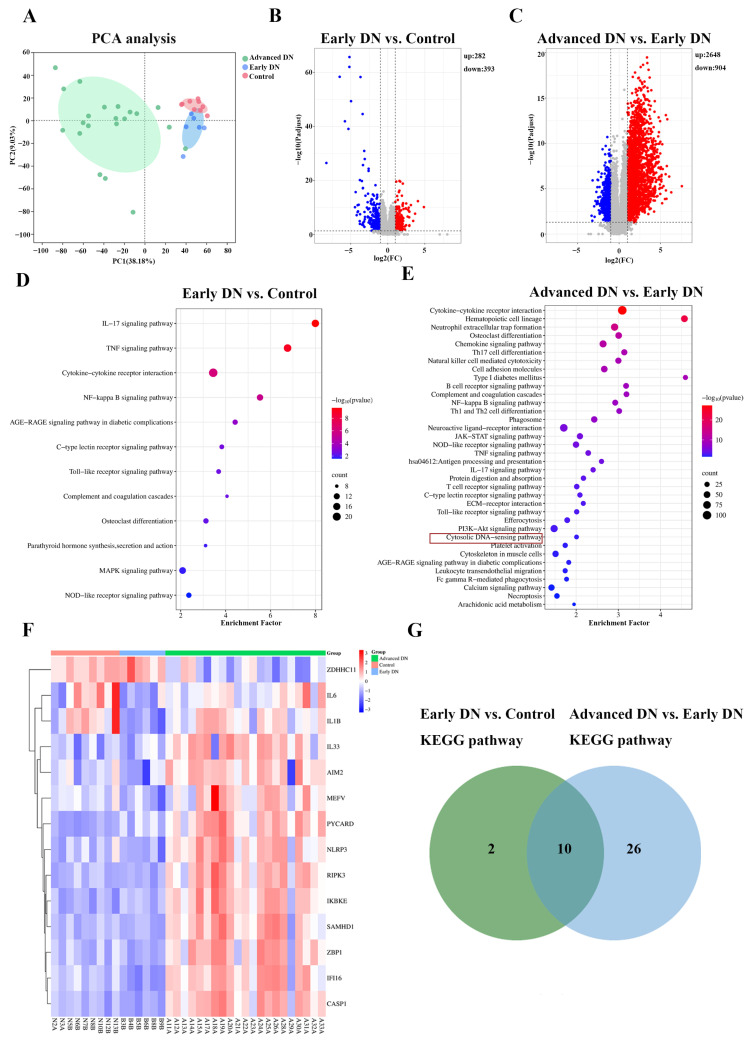
Analysis of the GSE142025 dataset. (**A**) The 2D PCA distribution trends of all groups. Green, blue, and pink ellipses represent the 95% confidence intervals for the advanced DN, early DN, and control groups, respectively. (**B**) Transcriptional profile differences between early DN and control visualized by volcano plot. (**C**) Transcriptional profile differences between advanced DN and early DN visualized by volcano plot.Red, blue, and gray dots represent significantly upregulated differentially expressed genes (DEGs), significantly downregulated DEGs, and non-significant DEGs, respectively. (**D**) KEGG pathways of differentially expressed gene (DEGs) in early DN vs. control. (**E**) KEGG pathways of DEGs in advanced DN vs. early DN (*p*-value < 0.05). The cytosolic DNA-sensing pathway (highlighted by a red box) is one of the pathways unique to advanced-stage DN. (**F**) Gene expression heatmap of the cytosolic DNA-sensing pathway. (**G**) Venn diagram showing 26 pathways unique to advanced-stage DN (*p*-value < 0.05).

The 2D PCA plot exhibited distinct separation trends among the control group, early DN group, and advanced DN group. Along principal component 1, the early DN group was positioned closer to the control group, whereas the advanced DN group was clearly separated from both (Figure 4A).

A threshold of *p*-adjust < 0.05 and |Log_2_FC| > 1 was set as the filter to obtain differentially expressed genes (DEGs). In the early DN vs. control comparison, 282 upregulated and 393 downregulated DEGs were identified (Figure 4B). In contrast, the advanced DN vs. early DN analysis yielded 2648 upregulated and 904 downregulated DEGs (Figure 4C).

KEGG pathway enrichment analysis of the DEGs revealed 12 DN-associated pathways in early DN vs. control (Figure 4D), including the IL-17, TNF, NF-κB, and AGE-RAGE signaling pathways. The advanced DN vs. early DN comparison identified 36 relevant pathways (Figure 4E), including cytokine–cytokine receptor interaction, osteoclast differentiation, and the cytosolic DNA-sensing pathway.

Using a Venn diagram we were able to identify 26 pathways that were unique to advanced-stage DN (Table S5, Figure 4G), notably the cytosolic DNA-sensing pathway, suggesting its key regulatory role in disease progression. A heatmap was used to visualize the 18 DEGs significantly associated with the cytosolic DNA-sensing pathway (Figure 4F).

### 2.8. Molecular Docking Analysis

The preliminary results showed that TNF was a common target in both PPI sub-networks and had the highest degree value. Moreover, TNF was also identified as a downstream target of the cytosolic DNA-sensing pathway. In the “component–target–disease” interaction network, salidroside (Sal), apigenin (Api), and tormentic acid (TA) were identified as potential ligands of TNF through database screening. Consequently, molecular docking analyses were performed to further investigate the interactions between TNF (PDB ID:4Y6O) and the aforementioned compounds.

**Figure 5:**
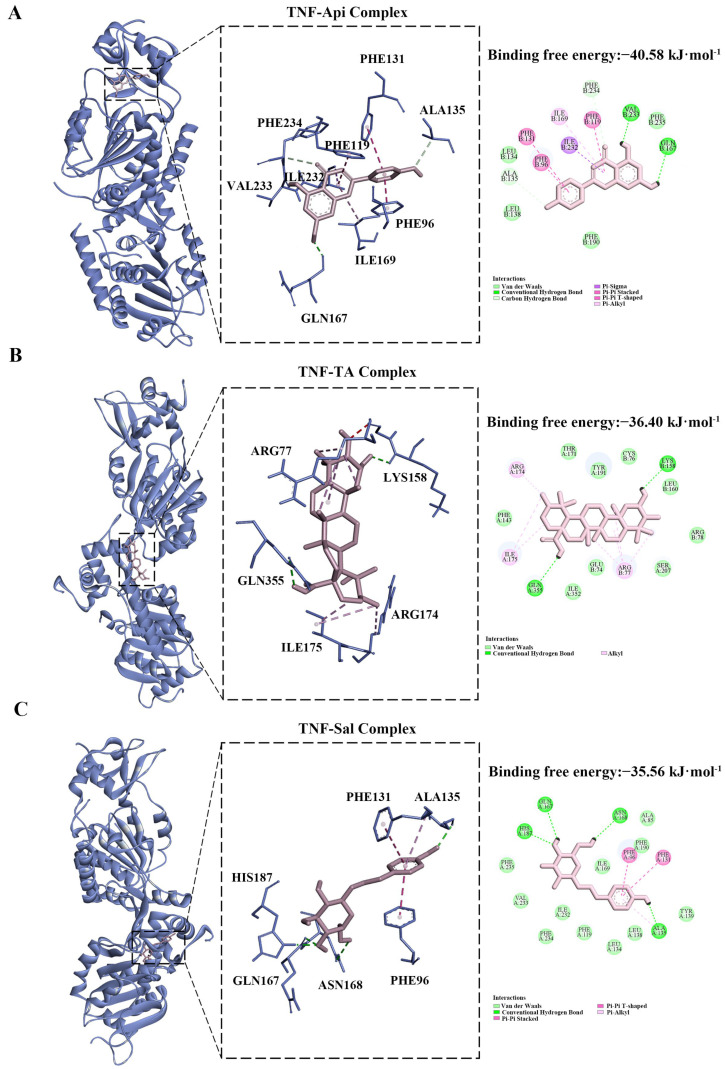
Molecular docking of molecule structures and binding sites between TNF and Api, TA, and Sal. The three small panels from left to right represent the overall docking conformation of the complex, the 3D visualization of the specific docking site and interaction forces, and the 2D visualization of the specific docking site and interaction forces, respectively. The dashed line represents the interaction between the molecule and the amino acid residue. (**A**) TNF-apigenin (Api) complex. (**B**) TNF-tormentic acid (TA) complex. (**C**) TNF-salidroside (Sal) complex.

The calculated binding free energies for the TNF-Api, TNF-TA, and TNF-Sal complexes were −40.58, −36.40, and −35.56 kJ·mol^−1^, respectively. These computational results suggested potential interactions between TNF and Api, TA, and Sal. The predicted binding modes were visualized using Discovery Studio 2019 (Figure 5).

### 2.9. Molecular Dynamics Analysis

Molecular dynamics simulations were employed to explore the structural dynamics of the ligand–receptor complexes. The three systems analyzed were the TNF-Api, TNF-Sal, and TNF-TA complexes.

**Figure 6:**
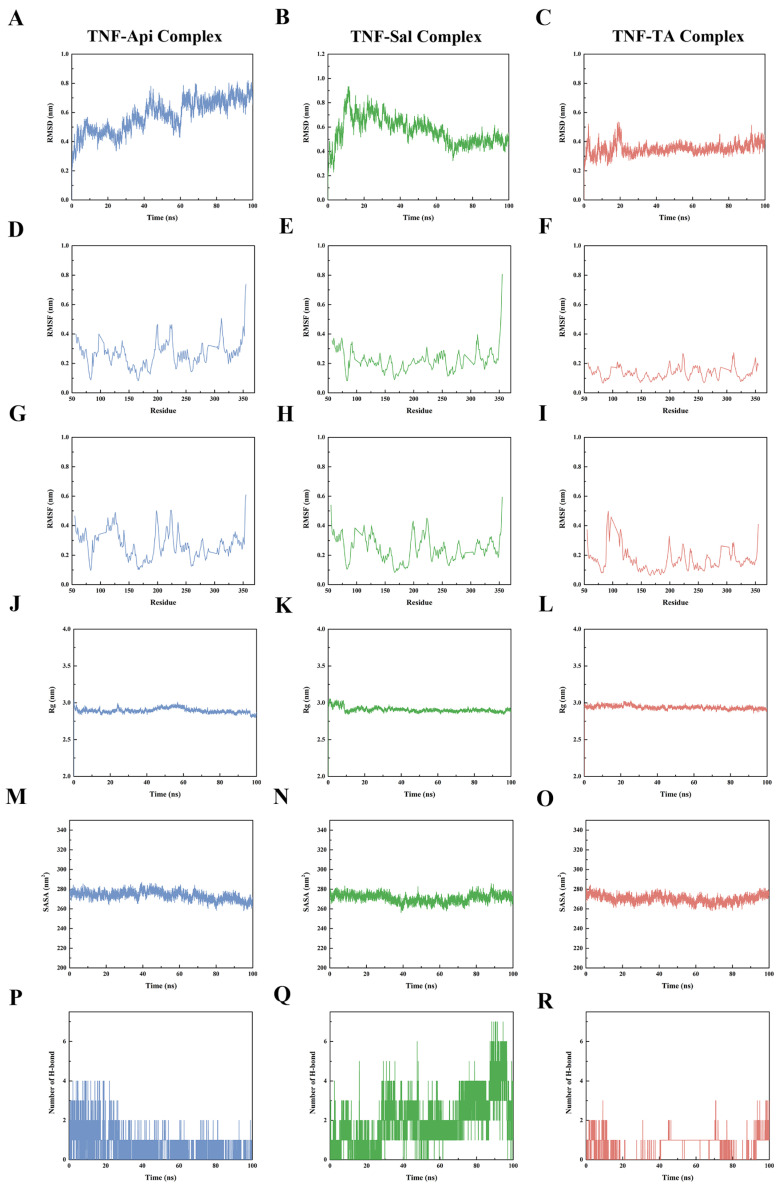
Molecular dynamics simulations of molecule structures and binding sites between TNF and Api, TA, and Sal. (**A**–**C**) RMSD curves of the three complexes. (**D**–**F**) RMSF curves of the three complexes (Chain A of TNF). (**G**–**I**) RMSF curves of the three complexes (Chain B of TNF). (**J**–**L**) Rg curves of the three complexes. (**M**–**O**) SASA curves of the three complexes. (**P**–**R**) Dynamic hydrogen bond count variations in the three compounds.

The root mean square deviation (RMSD) curves of TNF-Api and TNF-Sal stabilized after 70 ns (Figure 6A,B), whereas TNF-TA stabilized after 20 ns (Figure 6C), indicating conformational stabilization in all three systems.

Root mean square fluctuation (RMSF) analyses were performed for the A and B chains of TNF (PDB ID: 4Y6O). The average RMSF values for the TNF-Api, TNF-Sal, and TNF-TA complexes in chain A were 0.26 nm (Figure 6D), 0.22 nm (Figure 6E), and 0.13 nm (Figure 6F), respectively. In chain B, the values were 0.27 nm (Figure 6G), 0.25 nm (Figure 6H), and 0.17 nm (Figure 6I). These observations suggest reduced flexibility in the TNF-TA complex compared to the other systems.

The radius of gyration (Rg) curves of TNF-Api (Figure 6J), TNF-Sal (Figure 6K), and TNF-TA (Figure 6L) remained in narrow ranges of 2.80–3.04, 2.84–3.05, and 2.86–3.03 nm, respectively, indicating maintenance of overall structural compactness.

The solvent accessible surface area (SASA) curves of TNF-Api (Figure 6M), TNF-Sal (Figure 6N), and TNF-TA (Figure 6O) fluctuated stably within the ranges of 258.04–287.04, 256.18–286.76, and 284.40–258.11 nm^2^, reflecting stable protein-molecule binding.

The strong non-covalent interaction of hydrogen bonding was analyzed over time. The average number of hydrogen bonds formed by TNF-Api (Figure 6P), TNF-Sal (Figure 6Q), and TNF-TA (Figure 6R) was 0.54, 2.02, and 0.54, respectively, with maximum counts of 4, 7, and 3.

**Figure 7:**
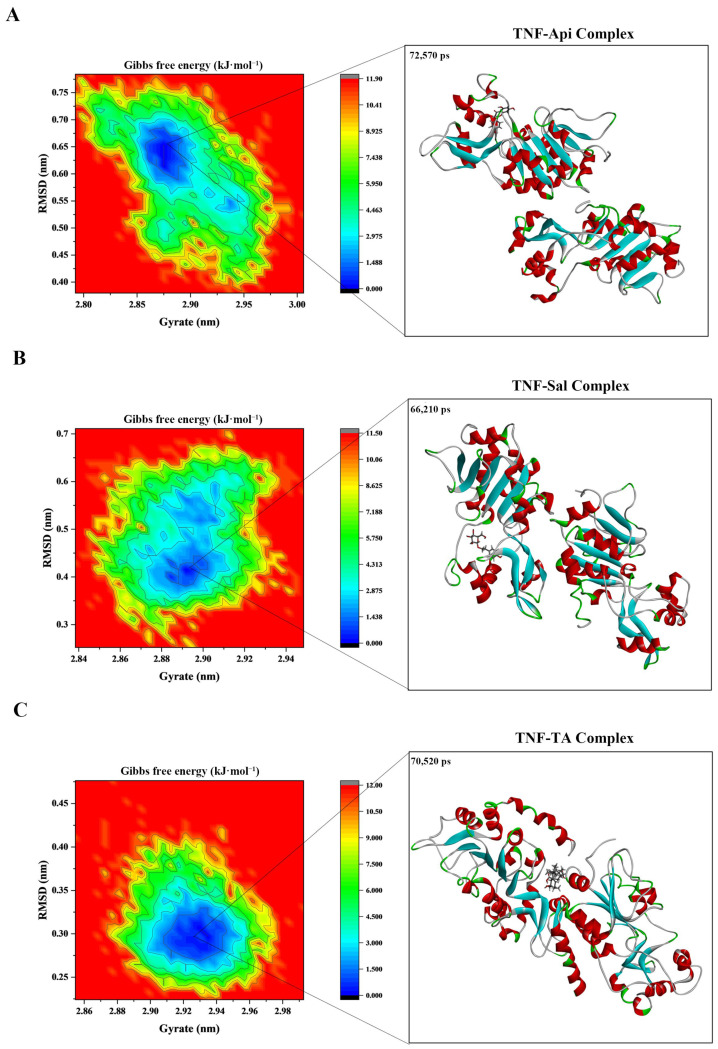
Molecular dynamics simulations of molecule structures and binding sites between TNF and Api, TA, and Sal. (**A**) TNF-Api complex. (**B**) TNF-TA complex. (**C**) TNF-Sal complex.

Gibbs energy landscape analysis provided insights into potential conformational transitions. RMSD and Rg were selected as collective variables to explore the energy landscapes of the systems. For the TNF-Api complex, a relatively stable region was observed, with Rg values of 2.86–2.90 nm and RMSD values of 0.60–0.67 nm (Figure 7A). The TNF-Sal complex showed an energy minimum at Rg 2.88–2.90 nm, with a corresponding RMSD of 0.37–0.45 nm (Figure 7B). For the TNF-TA complex, structural stability was observed when the RMSD and Rg values were 0.26–0.32 nm and 2.91–2.95 nm, respectively (Figure 7C).

Using the gmx_mmpbsa script, the estimated binding energies during equilibrium stages were calculated. The protein–ligand binding energies revealed favorable stability (Table S6), with values of −136.498 (TNF-Api), −34.250 (TNF-Sal), and −89.578 kJ·mol^−1^ (TNF-TA).

### 2.10. Effects of the LLF-Containing Serum on MPC5 Cell Viability

As shown in Table S7, incubation with 0.2 mM PA for 24 h resulted in approximately 50% cell viability in MPC5 cells, indicating an optimal modeling condition. As shown in Figure S3, treatment with 5%, 10%, and 20% LLF-containing serum for 24 h significantly increased cell viability in a dose-dependent manner. Therefore, these treatment conditions were selected for subsequent experiments.

### 2.11. The Protective Effect of LLF-Containing Serum on MPC5 Podocytes

**Figure 8:**
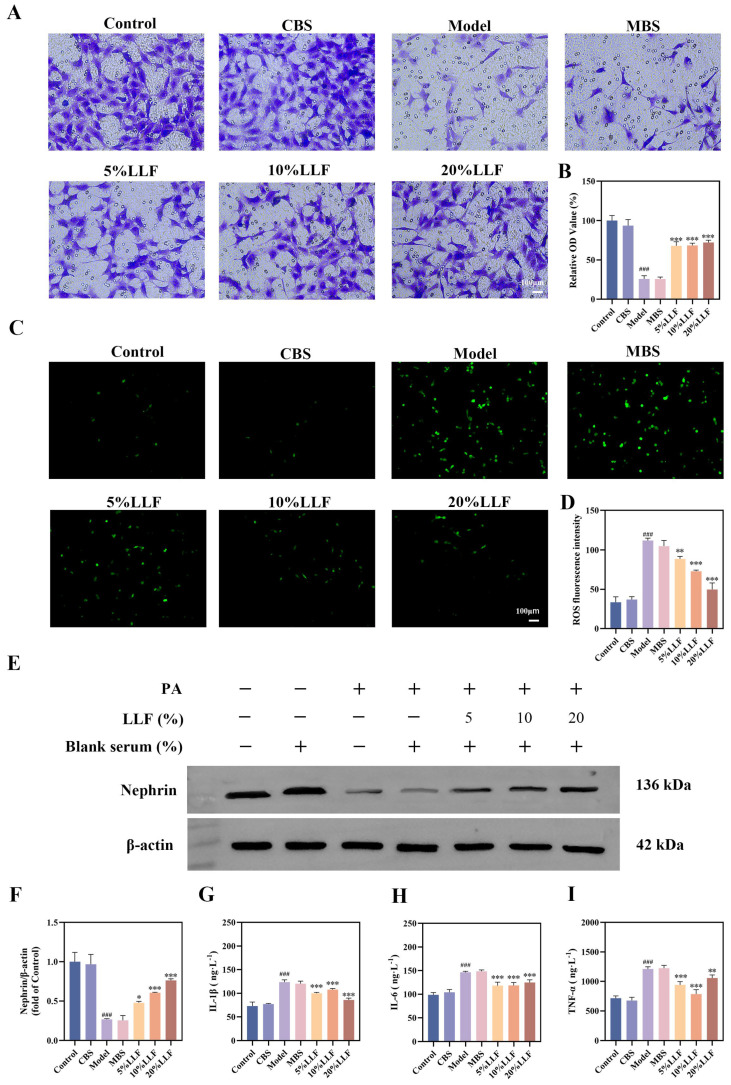
Podocyte-protective effect of LLF-containing serum. (**A**) Representative images of podocytes on the upper surface of the membranes in the Transwell system (scale bar: 100 µm). (**B**) Quantitative results of cell migration (*n* = 3). (**C**) Visualization of reactive oxygen species (ROS) (scale bar: 100 µm). (**D**) Quantification of ROS fluorescence intensity (*n* = 3). (**E**,**F**) Relative Nephrin protein expression levels (*n* = 3). (**G**) IL-6 concentration in podocyte culture supernatant (*n* = 3). (**H**) TNF-α concentration in podocyte culture supernatant (*n* = 3). (**I**) IL-1β concentration in podocyte culture supernatant (*n* = 3). ^###^ *p* < 0.001 vs. control group; * *p* < 0.05, ** *p* < 0.01, *** *p* < 0.001 vs. model group.

Podocyte injury is associated with impaired migratory capacity. In this context, using Transwell assays, we were able to show that there were significantly higher numbers of migrating cells in the treatment groups than in the model group (Figure 8A,B).

Elevated ROS levels are indicative of oxidative stress. To investigate whether LLF protects MPC5 podocytes via antioxidant mechanisms, ROS levels were assessed via flow cytometry and fluorescence microscopy. The results indicated suppression of PA-induced intracellular ROS levels by LLF-containing serum (Figure 8C,D).

The expression of Nephrin, a key podocyte marker protein, is reduced during podocyte injury. As shown in Figure 8E,F, treatment with 5%, 10%, and 20% LLF-containing serum upregulated Nephrin expression in a dose-dependent manner.

Inflammatory cytokine release contributes to a positive feedback loop exacerbating podocyte injury. Using ELISA, various concentrations of LLF-containing serum were shown to significantly suppress PA-induced IL-6, TNF-α, and IL-1β release (Figure 8G–I).

### 2.12. Modulation of the cGAS-STING Pathway by LLF-Containing Serum in MPC5 Podocytes

Integrated network pharmacology and Gene Expression Omnibus (GEO) database analyses identified the cytosolic DNA-sensing pathway as a pivotal mechanism through which LLF ameliorates DN. The cGAS-STING pathway as a key cytosolic DNA-sensing pathway component, is activated in PA-induced podocyte injury.

C176 specifically inhibits the cGAS-STING pathway by covalently binding to cysteine residue 91 within the STING transmembrane domain, thereby blocking activation-induced palmitoylation. Therefore, C176 served as the positive control in our experiment.

**Figure 9:**
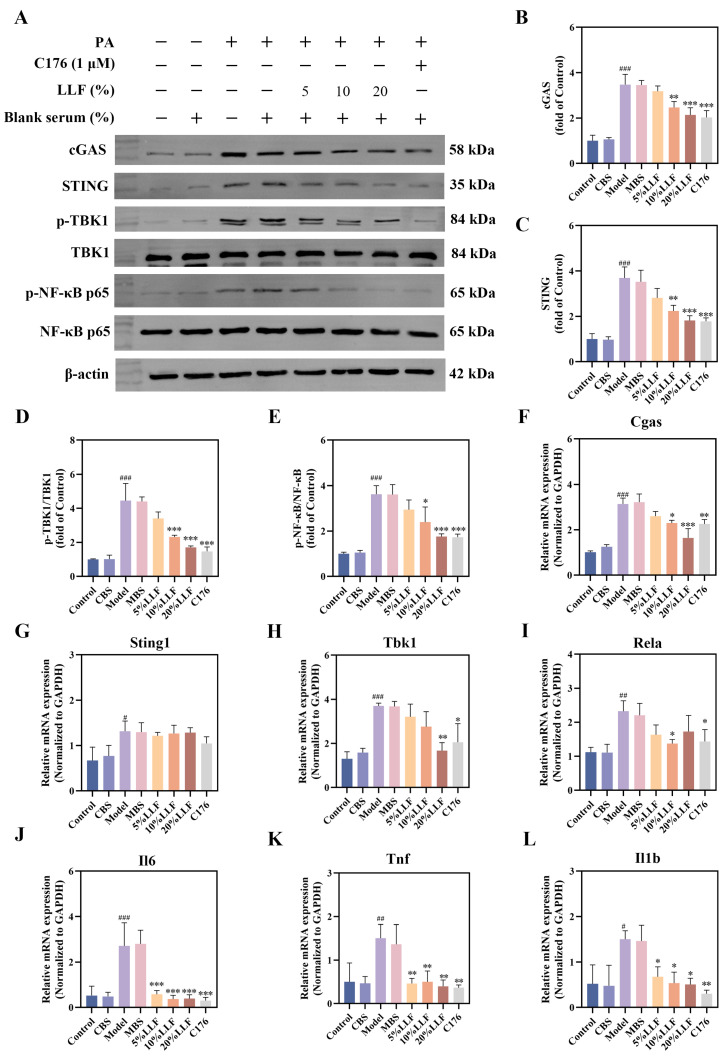
(**A**–**E**) Alterations in the relative expression levels of cGAS-STING pathway proteins in MPC5 podocytes (*n* = 3). (**F**) *Cgas* mRNA expression in MPC5 podocytes (*n* = 3). (**G**) *Sting1* mRNA expression in MPC5 podocytes (*n* = 3). (**H**) *Tbk1* mRNA expression in MPC5 podocytes (*n* = 3). (**I**) *Rela* mRNA expression in MPC5 podocytes (*n* = 3). (**J**) *Il6* mRNA expression in MPC5 podocytes (*n* = 3). (**K**) *Tnf* mRNA expression in MPC5 podocytes (*n* = 3). (**L**) *Il1b* mRNA expression in MPC5 podocytes (*n* = 3). ^#^ *p* < 0.05, ^##^ *p* < 0.01, ^###^ *p* < 0.001 vs. control group; * *p* < 0.05, ** *p* < 0.01, *** *p* < 0.001 vs. model group.

Western blot analysis revealed marked suppression of cGAS, STING, p-TBK1/TBK1, and p-NF-κB p65/NF-κB p65 expression following treatment with LLF-containing serum (10% and 20%) or C176 (1 μM) (Figure 9A–E). qRT-PCR analysis revealed a significant increase in the relative mRNA expression levels of *Cgas*, *Sting1*, *Tbk1*, *Rela*, *Il6*, *Tnf*, and *Il1b* upon PA stimulation, which was reversed by various concentrations of LLF-containing serum (Figure 9F–L). cGAS-STING signaling and NF-κB p65 exhibited coordinated expression patterns at both transcriptional and translational levels.

## 3. Discussion

LLF is an outstanding herb with the homology of medicine and food. When used as a single herb, studies have demonstrated that both raw and wine-steamed LLF extracts exhibit certain anti-DN activities, with the wine-steamed LLF extract showing superior efficacy compared to the raw LLF extract [19]. Furthermore, LLF is commonly employed in compound formulations of TCM. In the 2020 Edition of Chinese Pharmacopoeia, there are more than 100 prescriptions containing LLF [31]. Notably, LLF serves as a key component in several Chinese herbal formulations for DN treatment, including Keluoxin Capsule [32], Huangzhi Yishen Capsule [33], and Erzhi Pill [34]. Thus, the research and clinical applications of LLF demonstrate its potential as a therapeutic agent for DN.

According to network pharmacology analysis, LLF protected against DN via components such as Sal, Api, and TA. Based on SwissADME predictions, all three compounds showed high GI absorption and favorable drug-likeness (Figures S4–S6). Moreover, these compounds were identified as prototypes of LLF in rat plasma, suggesting their potential as primary bioactive constituents [35]. In terms of pharmacological activity research, Sal ameliorated DN through modulation of the TXNIP/NLRP3 [22], Akt/GSK-3β [36], Wnt/β-catenin [37], ERK1/2 phosphorylation [38], Src/Cav-1 [39], and AMPK/Sirt1 [40] pathways, demonstrating multi-target therapeutic effects. As a flavonoid with anti-DN activity [41], Api protected against DN through the MAPK/NF-κB/TNF-α [42], miR-423-5p-USF2 [43], and Nrf2/HO-1 [44] axes. In addition, TA exhibited antidiabetic, antioxidant, antihyperlipidemic, and anti-inflammatory activities [45], suggesting potential for DN treatment.

In the PPI analysis, we introduced a novel strategy by first pre-screening the entire network using topological importance metrics, followed by intersecting representative sub-networks to identify key targets. Applying this strategy revealed NFKB1, RELA, TNF, IL6, IL1B, TLR4, and CXCL8 as the primary targets for LLF in DN treatment. Gene expression meta-analyses revealed NFKB1 as a dominant transcriptional regulator in DN [46,47,48]. The NF-κB primary active subunit RELA (p65), which has pro-inflammatory functions, was characterized as a copper-related hub gene in DN progression [49,50]. The pathogenesis of DN is fundamentally driven by persistent subclinical inflammation [51]. TNF-α, IL-6, and IL-1β are key pro-inflammatory cytokines downstream of the NF-κB pathway that are encoded by TNF, IL6, and IL1B, respectively [52]. Preclinical evidence has indicated that TNF-α blockade using monoclonal antibodies, soluble receptor constructs, or pentoxifylline ameliorates renal dysfunction [53]. IL-6 signaling contributes to inflammatory responses [54,55]. Both the soluble IL-6R-mediated trans-signaling and the membrane-bound IL-6R-dependent classical signaling pathway contributed to the mechanisms underlying DN [56]. IL1B is a key genetic driver of renal fibrosis [57,58]. TLR4 recognizes damage-associated molecular patterns (DAMPs) to initiate inflammatory responses [59]. Upregulated TLR4 signaling translated diabetic metabolic alterations into kidney injury [60]. CXCL8 antagonism ameliorated glomerular damage in mice [61]. Moreover, urinary CXCL8 levels were markedly increased in type 2 diabetic nephropathy (T2DN) patients [62,63]. In conclusion, these core targets served as critical nodes for elucidating the regulatory mechanisms of LLF in DN.

Studies have shown that Api [42], Sal [19], and TA [64] all significantly decreased TNF-α levels in animal models. To further evaluate the potential interactions between these compounds and TNF, computational approaches including molecular docking and dynamics simulations were employed under static and dynamic conditions, respectively [65,66]. The TNF-Api, TNF-Sal, and TNF-TA complexes yielded binding scores of −40.58, −35.56, and −36.49 kJ·mol^−1^, respectively, with corresponding binding free energies of −136.498, −34.250, and −89.578 kJ·mol^−1^. Among these, the TNF-Api complex displayed the most favorable computational binding parameters, indicating a potentially critical role in the regulation of DN. Although computer-aided drug design (CADD) models, such as molecular docking and molecular dynamics simulations, have made significant contributions to predicting small molecule–protein interactions, they still suffer from limitations including insufficient accuracy of approximate computational models, the lack of suitable scoring functions and search algorithms, and an inability to fully represent realistic biological systems [67]. Therefore, the results of the molecular docking and dynamics simulations experiments should not be taken as the end result but rather as a good starting point for a deeper and more accurate analysis [68].

KEGG pathway enrichment of common targets facilitates the identification of drug regulatory mechanisms and disease-related networks [69]. Current research on the activation mechanisms of DN primarily focuses on metabolic dysfunction and low-grade inflammation, while the immunological activation mechanisms remain poorly studied [70]. Therefore, the significantly enriched immune-related pathways warrant further attention. Significantly enriched in KEGG analysis, the cytosolic DNA-sensing pathway triggers immune responses through pattern recognition receptor-mediated detection of microbial or self-dsDNA [71]. The cGAS-STING pathway is a pivotal component of the cytosolic DNA-sensing pathway. Since the initial identification of cGAS as a cytosolic DNA sensor in 2013, this pathway has remained a major research focus [72]. In this pathway, cGAS detects double-stranded DNA (dsDNA) and subsequently synthesizes the second messenger cGAMP. The generated cGAMP activates STING, initiating the production of interferons and pro-inflammatory cytokines [73]. Emerging evidence (since 2021) has demonstrated that the cGAS-STING pathway is critical in the regulatory mechanisms of DN [74,75]. Meanwhile, the GEO dataset analysis showed significant enrichment of the cytosolic DNA-sensing pathway in advanced-stage DN samples. Therefore, therapeutic targeting of the cytosolic DNA-sensing pathway, particularly the cGAS-STING axis, may represent a promising strategy for attenuating DN progression.

Podocyte injury directly contributes to DN pathogenesis, leading to renal functional decline [76]. Therefore, podocyte injury represents a critical therapeutic target in DN. Podocytes exhibit remarkable immunological competence, expressing both adaptive [77,78] and innate immune system components [70,79]. This immune-like phenotype suggests podocytes may function as nonhematopoietic antigen-presenting cells, participating in local immune responses that drive glomerular inflammation [80]. Studies have confirmed that both human and murine podocytes express all components of the cGAS-STING pathway at either the mRNA or protein level [80]. Importantly, STING activation was shown to damage podocytes in vitro, while cGAS-STING upregulation exacerbated renal injury in vivo [70,75]. Therefore, the cGAS-STING pathway serves as a critical mechanism of podocyte injury in DN.

Podocytes are particularly sensitive to saturated free fatty acids (FFAs) [81,82]. PA, a 16-carbon saturated FFA, can cause mitochondrial dysfunction and damage in MPC5 podocytes, thereby leading to mtDNA leakage into the cytosol through BAX-mediated macropore mechanisms [83]. Cytosolic mtDNA then activates the cGAS-STING-NF-κB pathway, which leads to inflammatory cytokine production [83]. The cGAS-STING and NF-κB pathways engage in a feed-forward loop: cytosolic DNA sensing triggers NF-κB-driven inflammation, which in turn amplifies STING signaling via mtDNA release [84].

**Figure 10:**
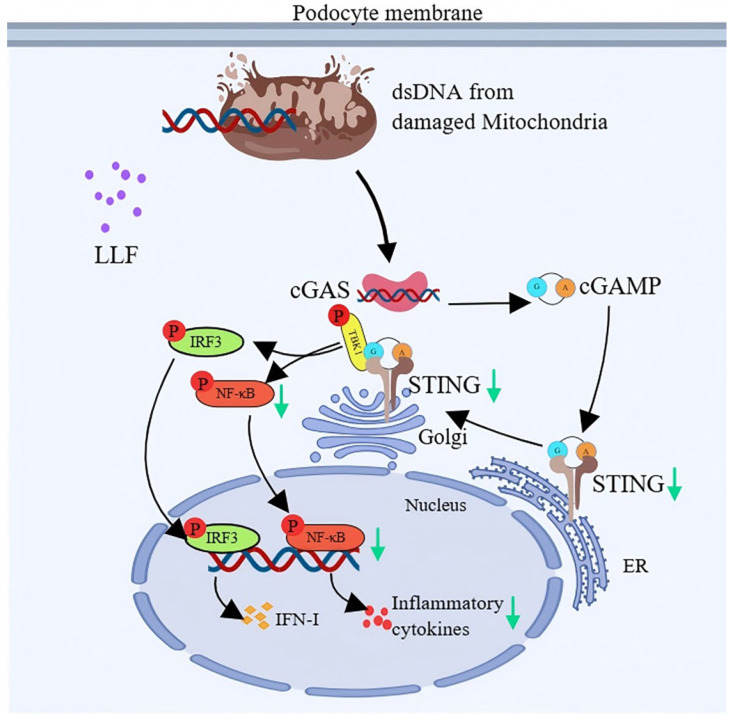
LLF exerts a podocyte-protective effect by inhibiting the cGAS-STING pathway.

Therefore, PA-induced injury was established in MPC5 podocytes prior to administration of LLF-containing serum. The protective effects of LLF were evaluated through cell migration assays, ROS detection, analysis of podocyte marker protein expression, and measurement of inflammatory cytokine levels. In parallel, network pharmacology predictions were validated by analyzing the expression of key proteins and genes within the cGAS-STING pathway. The results demonstrated that LLF alleviated PA-induced podocyte injury by inhibiting the cGAS-STING pathway (Figure 10).

In summary, this study indicated that LLF ameliorated DN through active components such as Sal, Api, and TA by affecting key targets including TNF, IL6, and RELA and modulating signaling pathways such as cGAS-STING and NF-κB.

## 4. Materials and Methods

### 4.1. Bioactive Component Identification and Target Gene Profiling in LLF

The TCMSP and HERB databases were used to profile the constituents of LLF. The SwissADME database was utilized to screen for active components with high gastrointestinal (GI) absorption and a drug-likeness score meeting at least four “yes” criteria. Subsequently, the TCMSP, SwissTargetPrediction, and HERB databases were employed to predict potential targets of the active compounds.

### 4.2. Screening of DN-Related Targets

Data from the OMIM, DrugBank, and GeneCards databases were accessed to identify DN-related targets via the keywords “diabetic nephropathy” and “diabetic kidney disease”. The retrieved disease targets from all three sources were merged to generate a union set.

### 4.3. Obtaining the Intersection Targets

The Venny tool was applied to visualize the overlapping targets between the active compounds of LLF and DN.

### 4.4. Constructing the PPI Network

Data from the STRING database were processed through Cytoscape 3.10.3 for PPI network construction. Subsequently, the MCODE and CytoHubba plugins were employed to perform topological analysis and identify potential core targets. In the network visualization, node colors represent the degree value, with darker colors indicating a higher degree.

### 4.5. Constructing a “Component–Target–Disease” Network

Based on the information regarding drugs, components, diseases, and targets, a “network” file and a “type” file were constructed. These files were imported into Cytoscape 3.10.3 to perform topological analysis of the “component–target–disease” network.

### 4.6. Functional Enrichment Analysis of GO and KEGG Pathways

Effective targets were functionally annotated using the DAVID database and subsequently visualized through the bioinformatics.com.cn platform.

### 4.7. GEO Analysis

The dataset GSE142025 was obtained from the GEO database. The RNA-seq data from GSE142025 were analyzed using the GEO2R online tool. The Supplementary File “GSE142025_RAW.tar” in GEO database was downloaded to retrieve gene expression levels. PCA was conducted based on the expression data. DEGs were subsequently visualized by employing volcano plots, KEGG enrichment maps, Venn diagrams, and heatmaps.

### 4.8. Molecular Docking

OpenBabel 2.4.1 software was applied to convert compound structures from SDF to MOL2 representations. The MOL2 file was energy-minimized using PyRx 0.8 software and transformed to the PDBQT format. Protein structures obtained from the PDB database were transformed to the PDBQT structural model using AutoDockTools 1.5.6. Compound and protein structures in the PDBQT format were docked using PyRx software. Discovery Studio 2019 was utilized to conduct visual processing on the specific binding sites in the docking results.

### 4.9. Molecular Dynamics

Protein–ligand interaction dynamics were studied through molecular dynamics simulations implemented in GROMACS 2020.3. A solvated system was prepared by embedding the complex in an SPC216 water model within a periodic boundary box (minimum 1.0 nm protein-to-edge distance). After adding Na^+^/Cl^−^ counterions to neutralize the system, sequential equilibration was performed under NVT (100 ps, 300 K) followed by NPT (100 ps, 1 bar) conditions prior to production molecular dynamics. Bond constraints were applied via the LINCS algorithm. Trajectory analysis and visualization were performed via VMD 1.9.3 and PyMOL 2.4.1. Binding free energies were computed using gmx_mmpbsa.

### 4.10. Preparation of LLF Extract

LLF samples were ground into fine powder, and 100 g aliquots were subjected to triple ultrasonic extraction with 1.5 L of 50% ethanol. The extracts were filtered, pooled, and concentrated via rotary evaporation at 50 °C, followed by freeze-drying.

### 4.11. Preparation of Drug-Containing Serum

SPF Biotechnology Co., Ltd. (Beijing, China) provided Sprague-Dawley rats (Seven-week-old, male, 200 ± 20 g). The rats were randomly allocated into a control group (receiving normal saline) and a treatment group (receiving LLF extract at 4.2 g·kg^−1^·d^−1^). All rats received daily oral gavage for one week. Following the last dose, all rats underwent terminal blood collection from the abdominal aorta. The whole-blood samples were stood for 2 h, followed by centrifugation at 4 °C (3000 rpm, 15 min) to harvest serum. In the cell drug treatment, the drug-containing serum was mixed with the culture medium at specific ratios (5%, 10%, and 20%).

### 4.12. Cell Culture

MPC5 cells (BeNa Culture Collection, Beijing, China) were cultured in DMEM (Gibco, Carlsbad, CA, USA) supplemented with 10% FBS (Mediatech, Manassas, VA, USA) and 1% penicillin–streptomycin (BasalMedia, Shanghai, China).

### 4.13. Modeling Concentration and Time Selection

Since elevated FFAs represent a key pathological feature of DN and podocytes exhibit high susceptibility to saturated fatty acids, PA was employed to induce injury in MPC5 podocytes.

MPC5 cells were treated with vehicle as a control or four different concentrations (0.1–0.4 mM) of PA. After incubation for 12, 24, or 48 h, the culture medium was discarded. Subsequently, CCK-8 reagent was added for absorbance measurement.

### 4.14. Optimization of Drug Concentration and Dosing Time

MPC5 cells were allocated to control, model, and treatment groups (cultured with complete medium containing 2.5%, 5%, 10%, 20%, or 30% LLF-containing serum). After pre-treatment, the model and treatment groups were stimulated with PA, whereas the control group received BSA solution. Upon completion of the modeling process, cell viability was assessed.

### 4.15. Cell Grouping and Treatment

According to the CCK-8 assay results, MPC5 cells were categorized into seven experimental groups: the control group, control group treated with complete medium containing 20% blank serum (CBS), model group, model group treated with complete medium containing 20% blank serum (MBS), 5% LLF group (cultured with complete medium containing 5% LLF-containing serum and 15% blank serum), 10% LLF group (cultured with complete medium containing 10% LLF-containing serum and 10% blank serum), and 20% LLF group (cultured with complete medium containing 20% LLF-containing serum). The CBS and MBS groups served as blank serum controls to exclude the potential influence of the serum itself on the cells. In theory, no statistically significant differences (*p* > 0.05) should be observed in any measured indicators when comparing the CBS vs. Control group or the MBS vs. Model group.

### 4.16. Cell Migration Experiment

MPC5 cells were seeded into Transwell chambers. After incubation, the membrane underwent 4% paraformaldehyde fixation prior to 0.1% crystal violet staining. Finally, the chambers were rinsed and air-dried. Cell migration was evaluated either by microscopic cell counting or by measuring absorbance at 750 nm.

### 4.17. Detection of Cellular ROS

Preparation of the DCFH-DA fluorescence probe working solution: The DCFH-DA fluorescent probe was diluted to prepare a 10 µM working solution.

Qualitative detection: After intervention, the culture medium was removed from each well, followed by PBS washing. Subsequently, cells were incubated with DCFH-DA working solution. Finally, microscopic imaging was performed.

Quantitative detection: After intervention, the cells in each well were digested, resuspended in the DCFH-DA fluorescent probe working solution, and then incubated at 37 °C in the dark for 20 min. Finally, the cells were centrifuged (1600 rpm, 5 min), washed with DMEM, and analyzed via flow cytometry.

### 4.18. Detection of Cellular Inflammatory Factors

After treatment, the cells in each well were digested and centrifuged (3000 rpm, 20 min) to collect the supernatant. The levels of IL-6, TNF-α, and IL-1β were quantified using manufacturer-specified assay kits.

### 4.19. qRT-PCR Analysis

The total RNA was reverse-transcribed in a PCR instrument (Eastwin Life Sciences Inc., Beijing, China). qRT-PCR was conducted with a fluorescence quantitative PCR system (Bio-Rad, Hercules, CA, USA) using PCR plates and sealing films. The relative expression levels of *Cgas*, *Sting1*, *Tbk1*, *Rela*, *Il6*, *Tnf*, and *Il1b* were analyzed by applying the 2^−ΔΔCT^ method. The specific primers for each gene are listed in Table 1.

### 4.20. Western Blot

After being stimulated with PA, MPC5 cells were centrifuged (12,000 rpm, 15 min, 4 °C) and the supernatant discarded. Cell lysates were obtained by incubating each group of cells with RIPA buffer (30 min). The protein concentrations of the cell lysis solutions were measured according to the BCA kit protocol. After 5 min of heating at 100 °C for denaturation, electrophoresis samples were generated that were composed of RIPA, 5 × loading buffer, and the lysis buffer. After SDS-PAGE separation, proteins were transferred to the PVDF membrane and incubated with the relevant primary and secondary antibodies. The specific dilution ratios and commercial sources (including manufacturer and catalog nomuber) of the primary antibody is stated in Table 2.

The results were visualized using the chemiluminescence imaging system visualized by ChemiScope 610. ImageJ v1.8.0 software was used for the quantitative detection of the subsequent band intensities.

### 4.21. Statistical Analysis

All statistical analyses were conducted employing GraphPad Prism 9.0 software. Data are presented as the mean ± standard deviation (SD).

## 5. Conclusions

Integrated with network pharmacology, molecular docking, molecular dynamics simulation, and in vitro pharmacological experiments, the anti-DN activity of LLF was systematically clarified. The results indicated that LLF exhibited therapeutic benefits against DN through a complex mechanism involving multiple components, targets, and signaling pathways. This study provides experimental evidence that LLF-containing serum protects podocytes by inhibiting the cGAS-STING pathway in MPC5 cells. These findings offer preliminary insights into the pharmacodynamic basis and underlying mechanisms of LLF’s effects in DN. However, the lack of in vivo validation limits the translational impact of our study. To address this limitation, future studies will utilize db/db mice as a spontaneous T2DN model to evaluate the renoprotective effects of LLF extract through long-term intragastric administration. The experimental protocol will include renal function evaluation, glucose and lipid metabolism profiling, renal histopathological analysis, podocyte ultrastructural observation, and analysis of cGAS-STING pathway protein expression.

## Figures and Tables

**Table 1:** Primer information.

Gene	Sense Primer (5′-3′)	Antisense Primer (5′-3′)
*Cgas*	TGAGGTCAATGAAGGGGTCGT	GAAGTGTTACAGCAGGGCTTCC
*Sting*	TCGGGTTTATTCCAACAGCG	GTTTAGCCTGCTCAAGCCGAT
*Tbk1*	GCAGTGCTAAGAAAGGACCATCA	TGCCTGAAGACCCTGAGAAAGAC
*Rela*	CGAGTCTCCATGCAGCTACG	TTTCGGGTAGGCACAGCAATA
*Il6*	GACTTCCATCCAGTTGCCTTCT	CTCATTTCCACGATTTCCCAGA
*Il1b*	AAATGCCACCTTTTGACAGTGA	AAAGAAGGTGCTCATGTCCTCATCC
*Tnf*	CCCTCACACTCACAAACCACC	CTTTGAGATCCATGCCGTTG
*GAPDH*	CCTCGTCCCGTAGACAAAATG	TGAGGTCAATGAAGGGGTCGT

**Table 2:** Detailed information on the primary antibody.

Primary Antibody	Species	Manufacturer	Catalog No.	Observed Molecular Weight	Dilution Ratio
Nephrin	Rabbit	Abcam	ab216692	136 kDa	1:1000
cGAS	Rabbit	Invitrogen	PA5-121188	58 kDa	1:1000
STING	Rabbit	Proteintech	80165-1-RR	35 kDa	1:1000
p-TBK1	Rabbit	Proteintech	82382-2-RR	84 kDa	1:1000
TBK1	Rabbit	Proteintech	28397-1-AP	84 kDa	1:1000
NF-κB p65	Rabbit	Abcam	ab16502	65 kDa	1:1000
p-NF-κB p65	Rabbit	Abcam	ab76302	65 kDa	1:1000
β-actin	Rabbit	Abcam	ab8227	42 kDa	1:5000

## Data Availability

No data was used for the research described in the article.

## Supplementary Materials

The following supporting information can be downloaded at: https://www.mdpi.com/article/10.3390/ijms26136303/s1.

## Abbreviations

The following abbreviations are used in this manuscript:

DN	Diabetic nephropathy
LLF	Ligustri Lucidi Fructus
TCM	Traditional Chinese Medicine
PA	Palmitic acid
Sal	Salidroside
Api	Apigenin
TA	Tormentic acid
GEO	Gene Expression Omnibus
CKD	Chronic kidney disease
ESKD	End-stage kidney disease
AGEs	Advanced Glycation End-products
ROS	Reactive oxygen species
PPI	Protein–protein interaction
GO	Gene Ontology
BP	Biological process
CC	Cellular component
MF	Molecular function
RMSD	Root mean square deviation
RMSF	Root mean square fluctuation
Rg	Radius of gyration
SASA	Solvent accessible surface area
DAMPs	Damage-associated molecular patterns
FFAs	Free fatty acids
T2DN	Type 2 diabetic nephropathy
GI	Gastrointestinal
CBS	Control group treated with blank serum
MBS	Model group treated with blank serum
SD	Standard deviation
ANOVA	Analysis of variance
CADD	Computer-aided drug design
