# Supplementary material for: Network Pharmacology, Molecular Dynamics Simulation, and Biological Validation Insights into the Potential of Ligustri Lucidi Fructus for Diabetic Nephropathy
Source: Int J Mol Sci. 2025 Jun 30;26(13):6303. doi: 10.3390/ijms26136303 (PMC12249564; doi:10.3390/ijms26136303)
Supplement: Supplementary file 1 [file ijms-26-06303-s001.zip › ijms-3708925-supplementary.pdf]

Supplementary materials

Table S1. Active compounds screened via the SwissADME database.

Chem CID	Compound Name	MF	GI absorption	Druglikeness				
				Lipinski	Ghose	Veber	Egan	Muegge
73193	Tormentic acid	$C_{30}H_{48}O_5$	High	Yes	No	Yes	Yes	Yes
439533	Taxifolin	$C_{15}H_{12}O_7$	High	Yes	Yes	Yes	Yes	Yes
159278	Salidroside	$C_{14}H_{20}O_7$	High	Yes	No	Yes	Yes	Yes
5280343	Quercetin	$C_{15}H_{10}O_7$	High	Yes	Yes	Yes	Yes	Yes
637520	Methylcinnamate	$C_{10}H_{10}O_2$	High	Yes	Yes	Yes	Yes	No
5280445	Luteolin	$C_{15}H_{10}O_6$	High	Yes	Yes	Yes	Yes	Yes
93009	Bornyl acetate	$C_{12}H_{20}O_2$	High	Yes	Yes	Yes	Yes	No
5280863	Kaempferol	$C_{15}H_{10}O_6$	High	Yes	Yes	Yes	Yes	Yes
637566	Geraniol	$C_{10}H_{18}O$	High	Yes	No	Yes	Yes	No
3314	Eugenol	$C_{10}H_{12}O_2$	High	Yes	Yes	Yes	Yes	No
440735	Eriodictyol	$C_{15}H_{12}O_6$	High	Yes	Yes	Yes	Yes	Yes
3026	DBP	$C_{16}H_{22}O_4$	High	Yes	Yes	Yes	Yes	Yes
5366074	Damascenone	$C_{13}H_{18}O$	High	Yes	Yes	Yes	Yes	No
5281708	Daidzein	$C_{15}H_{10}O_4$	High	Yes	Yes	Yes	Yes	Yes
5280372	Coniferin	$C_{16}H_{22}O_8$	High	Yes	No	Yes	Yes	Yes
65575	Cedrol	$C_{15}H_{26}O$	High	Yes	No	Yes	Yes	No
689043	Caffeic acid	$C_9H_8O_4$	High	Yes	Yes	Yes	Yes	No
24114	Butyl butyryllactate	$C_{11}H_{20}O_4$	High	Yes	Yes	Yes	Yes	Yes
5280443	Apigenin	$C_{15}H_{10}O_5$	High	Yes	Yes	Yes	Yes	Yes
6999980	Linalyl acetate	$C_{12}H_{20}O_2$	High	Yes	Yes	Yes	Yes	No

Table S2. Data on active ingredients in the "Component-Target-Disease" network.

Compound Name	Degree	Betweenness Centrality	Closeness Centrality	Average Shortest Path Length
Quercetin	138	0.0490	0.4410	2.2673
Apigenin	129	0.0378	0.4325	2.3119
Luteolin	113	0.0240	0.4182	2.3911
Kaempferol	103	0.0217	0.4097	2.4406
Daidzein	101	0.0246	0.4081	2.4505
DBP	56	0.0097	0.3734	2.6782
Butyl butyryllactate	53	0.0085	0.3720	2.6881
Caffeic acid	48	0.0064	0.3686	2.7129
Taxifolin	43	0.0023	0.3653	2.7376

Tormentic acid	39	0.0049	0.3620	2.7624
Eugenol	37	0.0039	0.3607	2.7723
Geraniol	34	0.0039	0.3594	2.7822
Eriodictyol	33	0.0031	0.3588	2.7871
Linalyl acetate	25	0.0020	0.3538	2.8267
Salidroside	25	0.0019	0.3538	2.8267
Cedrol	17	0.0009	0.3489	2.8663
Methylcinnamate	16	0.0007	0.3483	2.8713
Damascenone	14	0.0001	0.3471	2.8812
Bornyl acetate	10	0.0001	0.3447	2.9010
Coniferin	7	0.0001	0.3430	2.9158

Table S3. Annotation of KEGG pathways.

Term ID	Description	Count	*P* value	Gene Ratio	Genes
hsa04933	AGE-RAGE signaling pathway in diabetic complications	45	1.41×10^{-34}	0.0118	CXCL8, SERPINE1, PIK3CD, PIK3CB, PIK3R1, TNF, RELA, ICAM1, THBD, MAPK9, MAPK8, CCND1, CASP3, PIM1, CCL2, AKT1, MAPK1, RAC1, JAK2, MAPK3, JUN, VCAM1, PRKCB, STAT1, MMP2, PRKCD, STAT3, PRKCA, MAPK14, SELE, F3, TGFBR1, NFKB1, VEGFA, MAPK10, COL1A1, IL1A, COL3A1, IL6, PIK3CA, IL1B, BCL2, AGTR1, BAX, NOX4

hsa04668	TNF signaling pathway	40	3.51×10^{-25}	0.0139	CEBPB, PIK3CD, PIK3CB, PIK3R1, PTGS2, TNF, RELA, ICAM1, IKBKB, MAPK9, CASP7, MAPK8, CASP8, CASP3, CCL2, AKT1, MAPK1, IKBKG, MAPK3, JUN, VCAM1, CHUK, IFNB1, MMP3, CFLAR, FOS, MAPK14, SELE, MMP9, NFKB1, TNFRSF1A, MAPK10, NFKBIA, CXCL10, ADAM17, IL6, PIK3CA, IRF1, IL1B, MAP3K14
hsa04210	Apoptosis	40	8.92×10^{-23}	0.0159	PIK3CD, PIK3CB, PIK3R1, TNF, RELA, ACTB, CASP9, IKBKB, MAPK9, CASP7, MAPK8, CASP8, CASP3, CTSK, AKT1, MAPK1, BAK1, IKBKG, CTSD, MCL1, MAPK3, CTSB, JUN, PARP1, CHUK, GADD45A, CFLAR, FOS, NFKB1, TNFRSF1A, MAPK10, NFKBIA, PIK3CA, BCL2, BAX, BIRC5, CYCS, RAF1, MAP3K14, BCL2L1

hsa04151	PI3K-Akt signaling pathway	63	$1.41×10^{-22}$	0.0424	CHRM2, GSK3B, CDKN1A, FLT1, HSP90AB1, FLT3, PIK3CD, PIK3CB, PIK3CG, IGF1R, GHR, CASP9, IKBKB, CCND2, CCND1, MYC, KDR, AKT1, IKBKG, RAC1, JAK2, JAK3, JAK1, HSP90AA1, SYK, CHUK, PRKCA, PIK3CA, CCNE1, KIT, RAF1, MET, TLR4, PIK3R1, EGFR, RELA, INS, RXRA, ERBB3, ERBB2, SPP1, MAPK1, MCL1, MAPK3, IFNB1, EGF, INSR, IGF2, NFKB1, IL2, PTK2, VEGFA, IL4, COL1A1, GH1, IL6, CDK6, RPS6KB1, CDK2, BCL2, MDM2, FGFR1, BCL2L1
hsa04657	IL-17 signaling pathway	34	$2.31×10^{-22}$	0.0111	GSK3B, CEBPB, CXCL8, HSP90AB1, PTGS2, TNF, RELA, IKBKB, MAPK9, MAPK8, CASP8, CASP3, CCL2, MAPK1, IKBKG, MAPK3, JUN, HSP90AA1, CHUK, MMP1, IL13, MMP3, FOS, MAPK14, MMP9, NFKB1, MAPK10, IL4, NFKBIA, CXCL10, IL6, IFNG, IL1B, LCN2
hsa04620	Toll-like receptor signaling pathway	36	$2.36×10^{-22}$	0.0128	CD86, CXCL8, PIK3CD, PIK3CB, PIK3R1, TNF, RELA, IKBKB, MAPK9, MAPK8, CASP8, CTSK, SPP1, AKT1, MAPK1, IKBKG, RAC1, JAK1, MAPK3, JUN, CHUK, IFNB1, STAT1, TYK2, FOS, MAPK14, NFKB1, MAPK10, NFKBIA, CXCL10, CXCL11, IL6, PIK3CA, IL1B, TLR9, TLR4

hsa04066	HIF-1 signaling pathway	32	4.57×10^{-18}	0.0129	CDKN1A, FLT1, PFKFB3, SERPINE1, PIK3CD, PIK3CB, PIK3R1, HIF1A, RELA, EGFR, IGF1R, INS, ERBB2, AKT1, HMOX1, MAPK1, EP300, MAPK3, NOS2, PRKCB, EGF, INSR, STAT3, PRKCA, NFKB1, VEGFA, IL6, IFNG, PIK3CA, RPS6KB1, BCL2, TLR4
hsa04010	MAPK signaling pathway	50	4.61×10^{-17}	0.0352	FLT1, FLT3, HSPB1, ELK1, TNF, IGF1R, IKBKB, PPP3CA, MYC, CASP3, KDR, AKT1, IKBKG, RAC1, PRKACA, CHUK, PRKCB, PLA2G4A, PRKCA, FOS, TGFBR1, TNFRSF1A, IL1A, RASA1, IL1B, KIT, MAPT, RAF1, MET, EGFR, RELA, INS, MAPK9, MAPK8, ERBB3, ERBB2, MAPK1, MAPK3, JUN, EGF, GADD45A, INSR, IGF2, MAPK14, NFKB1, NFKB2, VEGFA, MAPK10, MAP3K14, FGFR1
hsa04630	JAK-STAT signaling pathway	36	1.03×10^{-17}	0.0197	CDKN1A, PIK3CD, PIK3CB, PIK3R1, EGFR, GHR, CCND2, CCND1, MYC, PIM1, AKT1, EP300, JAK2, JAK3, JAK1, MCL1, IL10, IFNB1, EGF, STAT1, IL13, STAT3, PTPN11, TYK2, IL2, GFAP, IL4, GH1, IL6, IFNG, PIK3CA, BCL2, PTPN6, RAF1, PTPN2, BCL2L1

hsa04064	NF-kappa B signaling pathway	28	9.57×10^{-15}	0.0123	CXCL8, PTGS2, TNF, RELA, ICAM1, IKBKB, PLAU, IKBKG, VCAM1, SYK, PARP1, CHUK, PRKCB, GADD45A, CFLAR, NFKB1, TNFRSF1A, NFKB2, NFKBIA, CD40LG, LCK, IL1B, CSNK2B, BCL2, BTK, TLR4, MAP3K14, BCL2L1
hsa04370	VEGF signaling pathway	21	1.37×10^{-13}	0.0070	PRKCB, SRC, PLA2G4A, PIK3CD, HSPB1, PRKCA, PIK3CB, PIK3R1, PTGS2, MAPK14, PTK2, VEGFA, CASP9, PPP3CA, PIK3CA, KDR, AKT1, MAPK1, RAC1, RAF1, MAPK3
hsa04621	NOD-like receptor signaling pathway	31	1.88×10^{-10}	0.0221	CXCL8, HSP90AB1, TNF, RELA, IKBKB, MAPK9, MAPK8, CASP8, CCL2, MAPK1, IKBKG, JAK1, MAPK3, CTSB, JUN, HSP90AA1, CHUK, IFNB1, STAT1, PRKCD, TYK2, MAPK14, RHOA, NFKB1, MAPK10, NFKBIA, IL6, IL1B, BCL2, TLR4, BCL2L1
hsa04024	cAMP signaling pathway	33	9.25×10^{-10}	0.0265	CHRM2, PTGER2, PIK3CD, ADRB1, ADRB2, PIK3CB, PIK3R1, RELA, CRHR1, MAPK9, MAPK8, HTR6, EDNRA, ADORA1, AKT1, MAPK1, EP300, DRD1, RAC1, DRD2, PRKACA, MAPK3, JUN, FOS, RHOA, NFKB1, MAPK10, NFKBIA, PIK3CA, FXYD2, RAF1, PPARA, CFTR
hsa04930	Type II diabetes mellitus	15	3.66×10^{-9}	0.0055	INSR, PRKCD, PIK3CD, PIK3CB, PIK3R1, TNF, GCK, INS, MAPK10, IKBKB, MAPK9, MAPK8, PIK3CA, MAPK1, MAPK3

hsa04217	Necroptosis	26	7.97×10^{-9}	0.0186	HSP90AB1, PYGM, PYGL, TNF, MAPK9, MAPK8, CASP8, JAK2, JAK3, JAK1, HSP90AA1, PARP1, IFNB1, STAT1, STAT3, PLA2G4A, TYK2, CFLAR, TNFRSF1A, MAPK10, IL1A, IFNG, IL1B, BCL2, BAX, TLR4
hsa04622	RIG-I-like receptor signaling pathway	15	1.24×10^{-6}	0.0084	CXCL8, CHUK, IFNB1, MAPK14, TNF, RELA, NFKB1, MAPK10, NFKBIA, IKBKB, MAPK9, CXCL10, MAPK8, CASP8, IKBKG
hsa04150	mTOR signaling pathway	20	2.95×10^{-5}	0.0185	GSK3B, CHUK, PRKCB, INSR, PIK3CD, PRKCA, PIK3CB, PIK3R1, TNF, RHOA, INS, IGF1R, TNFRSF1A, IKBKB, PIK3CA, RPS6KB1, AKT1, MAPK1, RAF1, MAPK3
hsa04623	Cytosolic DNA-sensing pathway	14	1.54×10^{-4}	0.0097	CHUK, IFNB1, RELA, NFKB1, NFKBIA, IKBKB, CXCL10, TNF, CASP7, IL6, CASP8, IL1B, CASP3, IKBKG
hsa04152	AMPK signaling pathway	15	5.33×10^{-4}	0.0143	PFKFB3, INSR, PIK3CD, PIK3CB, PIK3R1, HMGCR, ACACA, INS, IGF1R, PIK3CA, CCND1, RPS6KB1, AKT1, PPARG, CFTR
hsa04310	Wnt signaling pathway	18	9.27×10^{-4}	0.0204	GSK3B, JUN, PRKCB, PRKCA, RHOA, MAPK10, MAPK9, PPP3CA, MAPK8, CCND2, APC, CCND1, MYC, CSNK2B, EP300, RAC1, PRKACA, PPARD

Table S4. Annotation of Gene Ontology (GO) function.

Category	Term ID	Description	Count	*P* value	Gene Ratio
BP	0009410	response to xenobiotic stimulus	45	7.24×10^{-29}	0.0131
BP	0006954	inflammatory response	54	3.97×10^{-27}	0.0222
BP	0043066	negative regulation of apoptotic process	58	8.10×10^{-27}	0.0264
BP	0010628	positive regulation of gene expression	56	3.09×10^{-26}	0.0251
BP	0045944	positive regulation of transcription by RNA polymerase II	82	2.83×10^{-23}	0.0619
BP	0006468	protein phosphorylation	40	4.72×10^{-22}	0.0145
BP	0043410	positive regulation of MAPK cascade	35	5.21×10^{-22}	0.0105
BP	0030335	positive regulation of cell migration	37	5.83×10^{-19}	0.0148
BP	0008284	positive regulation of cell population proliferation	47	4.12×10^{-18}	0.0263
BP	0006974	DNA damage response	25	1.42×10^{-8}	0.0161
CC	0005886	plasma membrane	190	1.06×10^{-21}	0.2692
CC	0043235	receptor complex	31	5.69×10^{-18}	0.0104
CC	0005576	extracellular region	102	2.10×10^{-17}	0.1112
CC	0005737	cytoplasm	108	2.39×10^{-17}	0.2887
CC	0009986	cell surface	49	4.07×10^{-16}	0.0318
CC	0070062	extracellular exosome	97	9.16×10^{-16}	0.1078
CC	0032991	protein-containing complex	46	1.62×10^{-13}	0.0332
CC	0005634	nucleus	167	1.17×10^{-8}	0.3003
CC	0005788	endoplasmic reticulum lumen	23	1.14×10^{-7}	0.0153
CC	0005739	mitochondrion	59	4.34×10^{-6}	0.0828
MF	0042802	identical protein binding	113	9.08×10^{-30}	0.0923
MF	0019899	enzyme binding	49	3.55×10^{-25}	0.0195
MF	0004879	nuclear receptor activity	21	9.16×10^{-21}	0.0028
MF	0004672	protein kinase activity	40	1.06×10^{-20}	0.0157
MF	0005515	protein binding	339	1.11×10^{-16}	0.7111
MF	0005496	steroid binding	15	2.21×10^{-16}	0.0016

MF	0020037	heme binding	26	7.17×10^{-16}	0.0081
MF	0042803	protein homodimerization activity	53	3.70×10^{-15}	0.0391
MF	0005524	ATP binding	78	3.21×10^{-14}	0.0803
MF	0019901	protein kinase binding	41	4.78×10^{-14}	0.0257

Table S5. 26 pathways uniquely enriched in advanced-stage diabetic nephropathy (DN)

Term ID	Description	Count	*P* value	Gene Ratio	Genes
hsa04640	Hematopoietic cell lineage	52	7.74×10^{-23}	0.0117	CSF3R, ITGAM, FLT3, ITGB3, IL5RA, CD3G, CD3E, CD3D, TNF, LOC102723407, CD38, HLA-DOA, HLA-DOB, CD33, HLA-DPA1, IL11, MME, ITGA4, FCER2, CD8B, CD8A, IL1B, MS4A1, HLA-DQB2, CD44, IL9R, HLA-DQB1, CSF1R, CD1E, CD1D, CD1C, CD1B, CSF2RA, CD1A, HLA-DMB, CD19, FCGR1A, HLA-DQA2, HLA-DQA1, HLA-DRB5, CD2, CD4, IL6, CD5, IL7, IL2RA, CD7, HLA-DPB1, HLA-DRA, IL7R, CD22, HLA-DRB1

hsa04613	Neutrophil extracellular trap formation	64	1.36×10^{-15}	0.0226	SIGLEC9, ITGAM, NCF1, NCF2, ITGB3, NCF4, ITGB2, H2AC15, ITGAL, MPO, H2AC16, H2AC17, H2AC11, H2AC12, H2AC13, FCGR3A, H2AC14, FCGR3B, H2BC26, LOC102723407, CASP4, CASP1, RAC2, CTSG, H3C8, FGA, VWF, PRKCB, FGG, CYBB, H2BC17, H2BC12, H2BC13, H2BC14, H2BC10, H4C13, H2BC11, TLR8, TLR7, PADI4, PLCB2, TLR2, SELPLG, C5AR1, FPR1, FPR3, FPR2, HDAC9, C3, H3C15, H3C13, CLEC7A, H3C14, H3C11, H3C12, H3C10, H3C2, FCGR1A, H3C7, ELANE, H2BC9, H2BC7, H4C9, FCGR2A
hsa04062	Chemokine signaling pathway	58	3.66×10^{-12}	0.0226	CXCL6, ITK, CXCL8, NCF1, CCL4L2, CXCL1, CXCL13, CXCL3, CXCL2, PIK3CG, CXCL5, RAC2, CCR7, CCR6, CCR5, JAK3, CCR4, PF4V1, CCR2, PRKCB, VAV1, FGR, HCK, XCL2, ELMO1, XCL1, DOCK2, PLCB2, CX3CR1, CCL13, CCL11, CCL3L3, WAS, CXCR4, ADCY8, RASGRP2, ADCY7, PIK3R6, PIK3R5, GNG2, CCL8, GNG4, CXCR3, CCL5, CCL4, CXCR2, CCL3, CCL2, CCL19, CCL18, CCL17, LYN, CCL24, XCR1, CCL22, CCL21, CCL20, PPBP

hsa04659	Th17 cell differentiation	39	7.36×10^{-11}	0.0424	IL23R, EBI3, CD3G, IL27, CD3E, IL2RG, CD3D, IL27RA, HLA-DMB, TBX21, IL21R, IL12RB1, HLA-DOA, JAK3, HLA-DOB, HLA-DQA2, HLA-DQA1, HLA-DPA1, JUN, HLA-DRB5, NFATC2, FOS, RUNX1, ZAP70, CD4, IL6, IFNG, IRF4, LCK, IL1B, IL2RA, IL2RB, HLA-DPB1, HLA-DRA, CD247, NFKBIE, HLA-DQB2, HLA-DRB1, HLA-DQB1
hsa04650	Natural killer cell mediated cytotoxicity	40	1.94×10^{-10}	0.0137	ITGB2, PRF1, FASLG, ITGAL, TNF, ICAM1, FCGR3A, KLRK1, LOC102723407, FCGR3B, KLRC4-KLRK1, TNFSF10, RAC2, KLRC1, MICB, IFNAR2, FCER1G, KLRC2, PRKCB, KLRC3, SH2D1A, NFATC2, GZMB, VAV1, NCR1, LAT2, ZAP70, NCR3, TYROBP, IFNG, LCK, RAET1L, FAS, CD48, PTPN6, LCP2, KLRD1, CD247, HCST, CD244

hsa04514	Cell adhesion molecules	48	2.28×10^{-10}	0.0185	NLGN3, CD86, SELPLG, ITGAM, CD80, ITGB2, ITGAL, CLDN1, ICAM1, SPN, HLA-DMB, CDH3, CTLA4, LRRC4C, ICOS, HLA-DOA, HLA-DOB, HLA-DQA2, HLA-DQA1, HLA-DPA1, CADM3, HLA-DRB5, NLGN4X, VCAM1, ITGA4, SELE, CD2, MAG, CLDN4, CD4, VCAN, PTPRC, CD40LG, SELL, CD6, CD8B, CD8A, CLDN9, HLA-DPB1, CD28, SDC1, HLA-DRA, PDCD1, SIGLEC1, CD22, HLA-DQB2, HLA-DRB1, HLA-DQB1
hsa04940	Type I diabetes mellitus	23	2.45×10^{-10}	0.0052	CD86, HLA-DRB5, GAD1, CD80, PRF1, GZMB, FASLG, TNF, HLA-DMB, IFNG, IL1B, HLA-DPB1, CD28, FAS, HLA-DRA, HLA-DOA, HLA-DOB, HLA-DQA2, HLA-DQA1, HLA-DQB2, HLA-DRB1, HLA-DPA1, HLA-DQB1
hsa04662	B cell receptor signaling pathway	33	1.83×10^{-9}	0.0107	BLK, LILRA6, IFITM1, DAPP1, LILRA1, LILRA2, LILRA4, LILRA5, CD79B, CD79A, LOC102723407, CD19, INPP5D, RAC2, LYN, JUN, CD72, PRKCB, NFATC2, LILRB1, LILRB2, FOS, LILRB3, LILRB4, LILRB5, VAV1, BANK1, BTK, PTPN6, FCGR2B, NFKBIE, CARD11, CD22

hsa04658	Th1 and Th2 cell differentiation	32	1.43×10^{-8}	0.0109	CD3G, CD3E, IL2RG, CD3D, HLA-DMB, TBX21, STAT4, IL12RB1, HLA-DOA, JAK3, HLA-DOB, HLA-DQA2, HLA-DQA1, HLA-DPA1, JUN, HLA-DRB5, NFATC2, FOS, RUNX3, ZAP70, CD4, IFNG, LCK, IL2RA, IL2RB, HLA-DPB1, HLA-DRA, CD247, NFKBIE, HLA-DQB2, HLA-DRB1, HLA-DQB1
hsa04145	Phagosome	44	3.17×10^{-8}	0.0123	ITGAM, NCF1, C1R, NCF2, ITGB3, NCF4, ITGB2, THBS2, MPO, CORO1A, FCAR, CTSS, C3, COMP, FCGR3A, LOC102723407, HLA-DMB, TUBA1A, FCGR3B, CLEC7A, TUBB3, MRC1, FCGR1A, HLA-DOA, HLA-DOB, HLA-DQA2, HLA-DQA1, HLA-DPA1, MSR1, HLA-DRB5, CYBB, MARCO, FCGR2A, CD209, HLA-DPB1, HLA-DRA, TLR6, RAB7B, FCGR2B, FCGR2C, HLA-DQB2, HLA-DRB1, HLA-DQB1, TLR2

hsa04080	Neuroactive ligand-receptor interaction	21	4.84×10^{-6}	0.0434	NPFFR2, GLP1R, PRSS1, OXTR, NPFFR1, GRIK4, HTR2A, GRM1, CYSLTR1, HTR7, UCN3, UCN2, C3AR1, BDKRB1, CTSG, QRFPR, PRSS2, TSHB, PTGDR, UTS2R, EDN1, EDN2, ADRA2A, TRHR, MTNR1A, UTS2, AVP, GIPR, CHRNA4, PTGER2, CHRNA6, C5AR1, PTAFR, FPR1, LPAR1, OPRL1, LPAR2, GPR83, FPR3, PLG, ADRB2, LPAR4, FPR2, MCHR1, CRHR1, KNG1, P2RY8, C3, HRH1, GRIN2A, CNR2, CNR1, KISS1R, GABRE, S1PR5, TAC1, S1PR4, DRD5, GABRQ, GABRP, P2RY13, P2RY10, GZMA, P2RY14, GCGR, HCRTR2, GRIN1, P2RX7, KISS1, P2RX5, P2RX1, PNOC
hsa04630	JAK-STAT signaling pathway	40	8.72×10^{-6}	0.0197	CDKN1A, CSF3R, IL23R, IL24, FHL1, IL5RA, CSF2RB, IL27, IL2RG, CSF2RA, IL27RA, SOCS3, CCND2, MYC, IL21R, STAT4, AOX1, IL12RB1, JAK3, IL10, IFNAR2, IL11, EGF, IL10RA, LIF, OSM, IL31RA, IL19, OSMR, IL6, IFNG, IL7, IFNE, IL2RA, IL2RB, IL9, PTPN6, IL7R, IL9R, CRLF2

hsa04612	Antigen processing and presentation	24	2.17×10^{-5}	0.0095	HLA-DRB5, KLRC2, KLRC3, KLRC4, HSPA6, TNF, CTSS, CD4, HLA-DMB, IFNG, CD8B, CD8A, HLA-DPB1, HLA-DRA, KLRD1, KLRC1, HLA-DOA, HLA-DOB, HLA-DQA2, HLA-DQA1, HLA-DQB2, HLA-DRB1, HLA-DPA1, HLA-DQB1
hsa04974	Protein digestion and absorption	26	2.30×10^{-4}	0.0055	PRSS1, COL15A1, COL16A1, COL14A1, COL11A1, ATP1A4, ATP1A3, KCNN4, PRSS2, CPA3, SLC6A19, SLC36A2, MME, COL22A1, SLC8A3, COL1A1, COL3A1, COL1A2, COL5A1, COL4A1, XPNPEP2, COL7A1, COL8A2, COL6A3, COL8A1, COL6A5
hsa04660	T cell receptor signaling pathway	28	4.66×10^{-4}	0.0186	ITK, CD3G, CD3E, CD3D, TNF, GRAP2, CTLA4, ICOS, IL10, JUN, NFATC2, FOS, VAV1, ZAP70, CD4, PTPRC, CD40LG, IFNG, CD8B, LCK, CD8A, CD28, PTPN6, LCP2, PDCD1, CD247, NFKBIE, CARD11
hsa04512	ECM-receptor interaction	22	8.06×10^{-4}	0.0104	LAMA2, VWF, ITGA4, ITGB3, LAMA3, TNC, FN1, LAMC2, HMMR, THBS2, COL1A1, COMP, COL1A2, COL4A1, TNN, CHAD, SPP1, COL6A3, SDC1, ITGB6, COL6A5, CD44

hsa04148	Efferocytosis	32	1.43×10^{-3}	0.0184	C1QB, CAMK2B, CX3CR1, C1QA, CEBPB, PTGER2, ITGB3, CAMK2A, ALOX15, SIRPG, STAB2, ATP2A3, ALOX5, STAB1, CASP1, HAVCR1, IL10, P2RY12, DUSP4, DUSP2, SPHK1, SIGLEC10, NFATC2, TIMD4, CPT1C, CH25H, AXL, ELMO1, PTPN6, RAB7B, XKR4, C1QC
hsa04151	PI3K-Akt signaling pathway	61	1.55×10^{-3}	0.0424	CDKN1A, CSF3R, FLT3, ITGB3, TNC, LAMC2, FASLG, PIK3CG, COMP, TCL1B, TCL1A, FGF7, LOC102723407, CCND2, FGF9, CREB3L3, TNN, MYC, MYB, NRTN, ITGB6, JAK3, IFNAR2, VWF, ITGA4, HGF, OSMR, COL4A1, COL6A3, COL6A5, TLR2, CSF1R, LAMA2, LAMA3, LPAR1, LPAR2, LPAR4, THBS2, IL2RG, PIK3R6, PIK3R5, GNG2, GNG4, CD19, CHAD, SPP1, PCK1, PCK2, EGF, OSM, FN1, G6PC1, COL1A1, IL6, COL1A2, LPAR5, IL7, IL2RA, IL2RB, IL7R, FGF10
hsa04623	Cytosolic DNA-sensing pathway	20	4.92×10^{-3}	0.0097	ZBP1, IL33, RIPK3, CCL4L2, CGAS, ZDHHC11, MEFV, SAMHD1, ZDHHC11B, PYCARD, IL6, AIM2, IFI16, IL1B, TNF, CCL5, CCL4, CASP1, NLRP3, IKBKE

hsa04611	Platelet activation	25	7.46×10^{-3}	0.0148	ITGB3, ADCY8, RASGRP2, ADCY7, PIK3R6, PIK3CG, PIK3R5, PTGS1, LYN, P2RY12, FGA, FCER1G, VWF, FGG, APBB1IP, COL1A1, COL3A1, FCGR2A, COL1A2, P2RX1, TBXAS1, BTK, LCP2, PLCB2, FERMT3
hsa04820	Cytoskeleton in muscle cells	40	7.70×10^{-3}	0.0272	LAMA2, COL11A1, ITGB3, FHL1, ATP1A4, FHL2, ATP1A3, FMN2, THBS2, ENO2, LMNB1, COMP, PDLIM1, PDLIM3, TNNI2, ITGB6, MYBPC1, MYBPC2, TPM4, CKM, ITGA4, FN1, AMPD1, COL1A1, COL3A1, FMNL1, VCAN, DIAPH3, COL1A2, COL5A1, COL4A1, TNNT1, MYL2, ZYX, COL6A3, SDC1, VIM, COL6A5, MYH7, FBN1
hsa04670	Leukocyte transendothelial migration	23	1.07×10^{-2}	0.0136	ITK, ITGAM, VCAM1, NCF1, ITGA4, PRKCB, NCF2, ACTN1, ITGB2, NCF4, CYBB, CXCR4, RHOH, ITGAL, CLDN1, MMP9, VAV1, ICAM1, CLDN4, CLDN9, RASSF5, MYL2, RAC2
hsa04666	Fc gamma R-mediated phagocytosis	20	1.49×10^{-2}	0.0116	LYN, MARCKSL1, NCF1, PRKCB, SPHK1, ARPC1B, WAS, VAV1, HCK, FCGR3A, MARCKS, FCGR2A, FCGR3B, LOC102723407, PTPRC, INPP5D, RAC2, FCGR1A, FCGR2B, PLPP2

hsa04020	Calcium signaling pathway	41	2.00×10^{-2}	0.0298	CAMK2B, OXTR, PTAFR, CAMK2A, ATP2A3, CXCR4, CALML3, MST1R, ADRB2, HTR2A, ADCY8, CACNA1E, ADCY7, CACNA1H, GRM1, CACNA1I, HRH1, FGF7, GNA15, GRIN2A, CYSLTR1, LOC102723407, HTR7, FGF9, BDKRB1, CD38, DRD5, PRKCB, EGF, HGF, SPHK1, NFATC2, GRIN1, TRHR, SLC8A3, P2RX7, P2RX5, P2RX1, CAMK4, PLCB2, FGF10
hsa04217	Necroptosis	28	2.11×10^{-2}	0.0186	CAMK2B, CAMK2A, ALOX15, TNFAIP3, FASLG, H2AC15, H2AC16, TNF, H2AC17, H2AC11, PYCARD, H2AC12, H2AC13, H2AC14, CASP1, TNFSF10, STAT4, NLRP3, JAK3, ZBP1, IFNAR2, IL33, RIPK3, CYBB, IFNG, IL1B, FAS, BIRC3
hsa00590	Arachidonic acid metabolism	14	2.31×10^{-2}	0.0074	PLA2G2D, PLA2G2A, CYP4F3, ALOX15, PLA2G3, ALOX15B, PLB1, PTGS1, HPGDS, CYP2B6, ALOX5, TBXAS1, CYP2E1, PTGDS

Table S6. Binding energy of the complex

Energy(KJ/mol)	TNF-Api	TNF-Sal	TNF-TA
Van der Waals Energy	-213.655	-116.144	-152.388
Electrostatic energy	-7.848	-55.958	-54.271
Polar solvation energy	110.376	158.321	135.446
Nonpolar solvation Energy	-25.371	-20.469	-18.366
Total Binding Energy	-136.498	-34.250	-89.578

Table S7. Effects of different modeling conditions on MPC5 cell viability. (Mean ± SD, *n* = 3)

Time (h)	Cell survival rate (%)			
	0.1 mM PA	0.2 mM PA	0.3 mM PA	0.4 mM PA
12	0.87 ± 0.04	0.69 ± 0.08	0.65 ± 0.09	0.62 ± 0.10
24	0.77 ± 0.06	0.54 ± 0.12	0.47 ± 0.06	0.34 ± 0.07
48	0.68 ± 0.06	0.34 ± 0.06	0.28 ± 0.04	0.24 ± 0.09

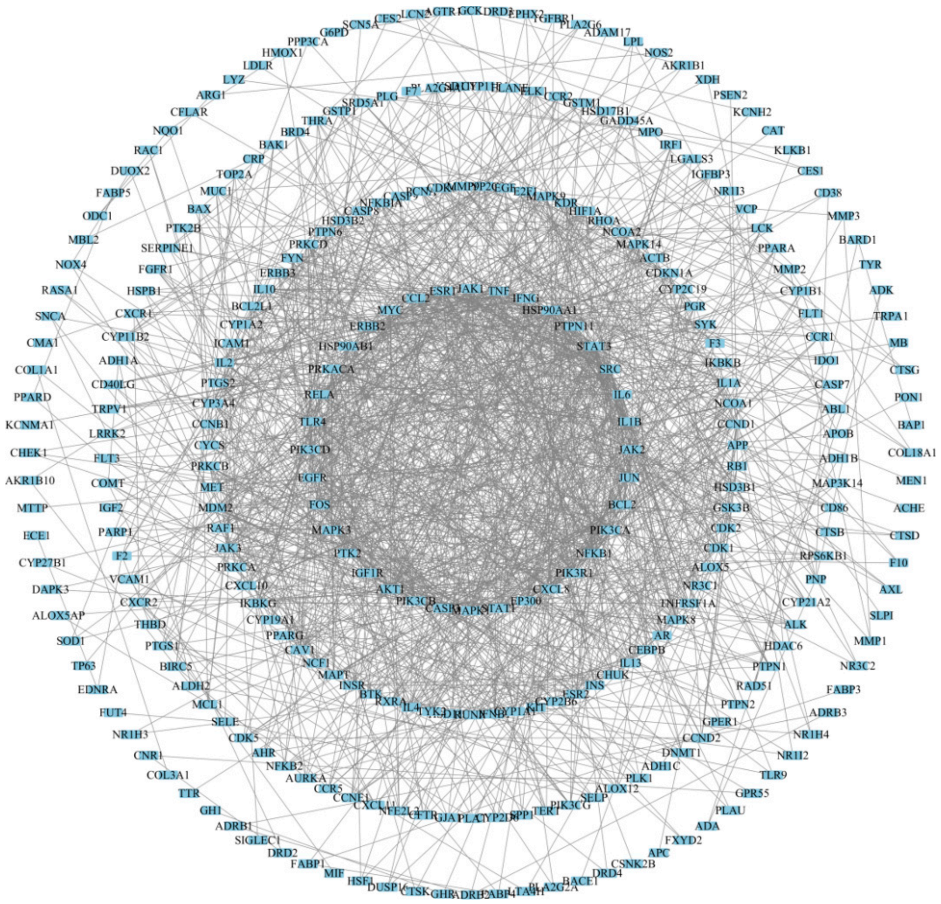

Figure S1. Overall Protein-protein interaction (PPI) network

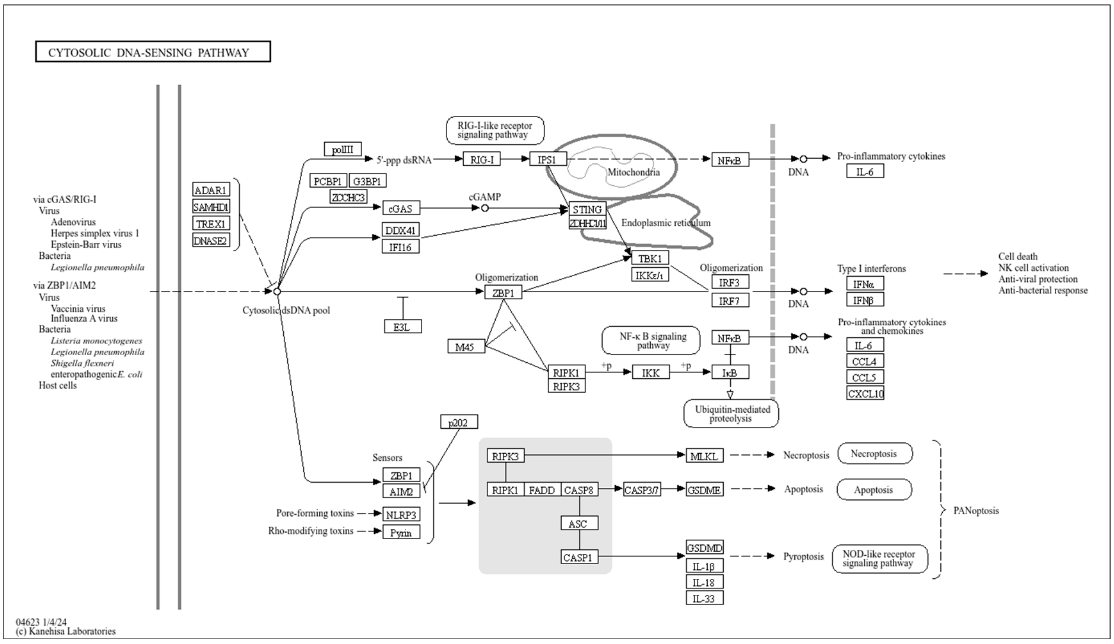

Figure S2. Map of the cytosolic DNA-sensing pathway in KEGG database

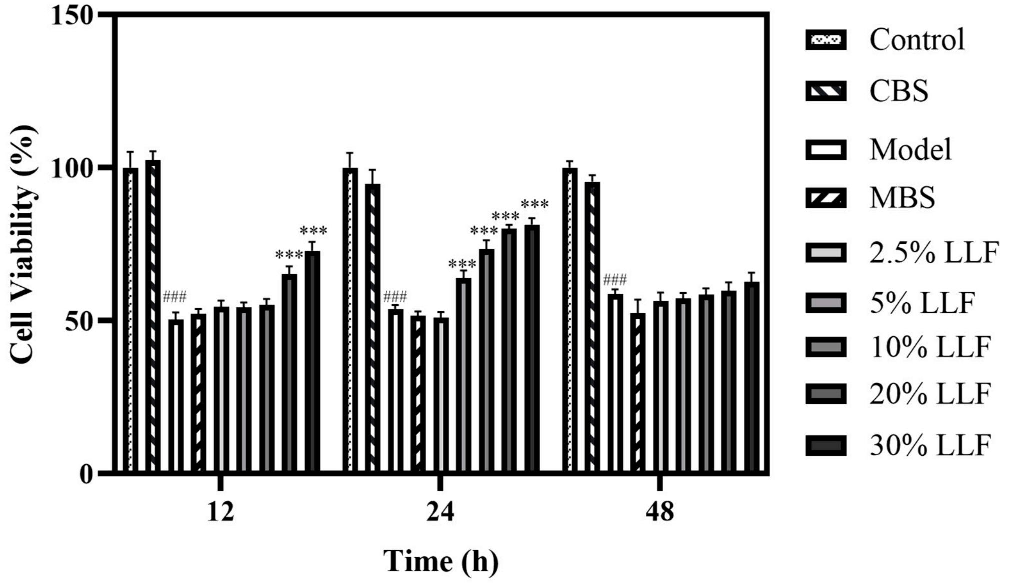

Figure S3. The cell viability under different drug concentrations and treatment durations. (n = 3). $^{\#}P < 0.05$, $^{\#\#}P < 0.01$, $^{\#\#\#}P < 0.001$ vs. Control group; $^{*}P < 0.05$, $^{**}P < 0.01$, $^{***}P < 0.001$ vs. Model group.

Molecule 1

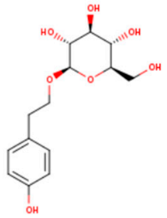

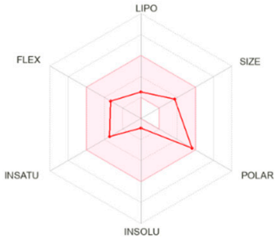

SMILES OC[C@H]1O[C@@H](OCCc2ccc(cc2)O)[C@@H]([C@H]([C@@H]1O)O)O

Physicochemical Properties	
Formula	C14H20O7
Molecular weight	300.30 g/mol
Num. heavy atoms	21
Num. arom. heavy atoms	6
Fraction Csp3	0.57
Num. rotatable bonds	5
Num. H-bond acceptors	7
Num. H-bond donors	5
Molar Refractivity	71.78
TPSA	119.61 Å²
**Lipophilicity**	
Log $P_{o/w}$ (iLOGP)	1.04
Log $P_{o/w}$ (XLOGP3)	-1.05
Log $P_{o/w}$ (WLOGP)	-1.25
Log $P_{o/w}$ (MLOGP)	-1.22
Log $P_{o/w}$ (SILICOS-IT)	-0.48
Consensus Log $P_{o/w}$	-0.59

Water Solubility	
Log *S* (ESOL)	-0.92
Solubility	3.60e+01 mg/ml ; 1.20e-01 mol/l
Class	Very soluble
Log *S* (Ali)	-0.97
Solubility	3.19e+01 mg/ml ; 1.06e-01 mol/l
Class	Very soluble
Log *S* (SILICOS-IT)	-0.44
Solubility	1.08e+02 mg/ml ; 3.61e-01 mol/l
Class	Soluble
**Pharmacokinetics**	
GI absorption	High
BBB permeant	No
P-gp substrate	No
CYP1A2 inhibitor	No
CYP2C19 inhibitor	No
CYP2C9 inhibitor	No
CYP2D6 inhibitor	No
CYP3A4 inhibitor	No
Log K_p (skin permeation)	-8.88 cm/s
**Druglikeness**	
Lipinski	Yes; 0 violation
Ghose	No; 1 violation: WLOGP<-0.4
Veber	Yes
Egan	Yes
Muegge	Yes
Bioavailability Score	0.55
**Medicinal Chemistry**	
PAINS	0 alert
Brenk	0 alert
Leadlikeness	Yes
Synthetic accessibility	4.26

Figure S4. Prediction of salidroside (Sal) properties based on the SwissADME database

Molecule 1

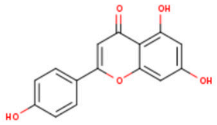

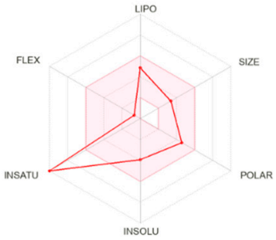

SMILES Oc1ccc(cc1)c1cc(=O)c2c(o1)cc(cc2O)O

Physicochemical Properties	
Formula	C15H10O5
Molecular weight	270.24 g/mol
Num. heavy atoms	20
Num. arom. heavy atoms	16
Fraction Csp3	0.00
Num. rotatable bonds	1
Num. H-bond acceptors	5
Num. H-bond donors	3
Molar Refractivity	73.99
TPSA	90.90 Å²

Lipophilicity	
Log $P_{o/w}$ (iLOGP)	1.89
Log $P_{o/w}$ (XLOGP3)	3.02
Log $P_{o/w}$ (WLOGP)	2.58
Log $P_{o/w}$ (MLOGP)	0.52
Log $P_{o/w}$ (SILICOS-IT)	2.52
Consensus Log $P_{o/w}$	2.11

Water Solubility	
Log *S* (ESOL)	-3.94
Solubility	3.07e-02 mg/ml ; 1.14e-04 mol/l
Class	Soluble
Log *S* (Ali)	-4.59
Solubility	6.88e-03 mg/ml ; 2.55e-05 mol/l
Class	Moderately soluble
Log *S* (SILICOS-IT)	-4.40
Solubility	1.07e-02 mg/ml ; 3.94e-05 mol/l
Class	Moderately soluble

Pharmacokinetics	
GI absorption	High
BBB permeant	No
P-gp substrate	No
CYP1A2 inhibitor	Yes
CYP2C19 inhibitor	No
CYP2C9 inhibitor	No
CYP2D6 inhibitor	Yes
CYP3A4 inhibitor	Yes
Log K_p (skin permeation)	-5.80 cm/s

Druglikeness	
Lipinski	Yes; 0 violation
Ghose	Yes
Veber	Yes
Egan	Yes
Muegge	Yes
Bioavailability Score	0.55

Medicinal Chemistry	
PAINS	0 alert
Brenk	0 alert
Leadlikeness	Yes
Synthetic accessibility	2.96

Figure S5. Prediction of apigenin (Api) properties based on the SwissADME database

Molecule 1

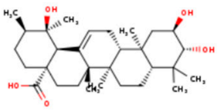

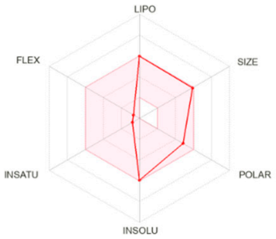

SMILES O[C@@H]1C[C@@]2(C)[C@H](C([C@H]1O)(C)C)CC[C@@]1([C@@H]2CC=C2[C@@]1(C)CC[C@@]1([C@H]2[C@](C)(O)[C@H](C)CC1)C(=O)O)C

Physicochemical Properties	
Formula	C30H48O5
Molecular weight	488.70 g/mol
Num. heavy atoms	35
Num. arom. heavy atoms	0
Fraction Csp3	0.90
Num. rotatable bonds	1
Num. H-bond acceptors	5
Num. H-bond donors	4
Molar Refractivity	139.28
TPSA	97.99 Å²

Lipophilicity	
Log $P_{o/w}$ (iLOGP)	3.31
Log $P_{o/w}$ (XLOGP3)	4.96
Log $P_{o/w}$ (WLOGP)	5.18
Log $P_{o/w}$ (MLOGP)	4.14
Log $P_{o/w}$ (SILICOS-IT)	4.07
Consensus Log $P_{o/w}$	4.33

Water Solubility	
Log *S* (ESOL)	-5.93
Solubility	5.76e-04 mg/ml ; 1.18e-06 mol/l
Class	Moderately soluble
Log *S* (Ali)	-6.76
Solubility	8.57e-05 mg/ml ; 1.75e-07 mol/l
Class	Poorly soluble
Log *S* (SILICOS-IT)	-4.48
Solubility	1.63e-02 mg/ml ; 3.33e-05 mol/l
Class	Moderately soluble

Pharmacokinetics	
GI absorption	High
BBB permeant	No
P-gp substrate	Yes
CYP1A2 inhibitor	No
CYP2C19 inhibitor	No
CYP2C9 inhibitor	No
CYP2D6 inhibitor	No
CYP3A4 inhibitor	No
Log K_p (skin permeation)	-5.76 cm/s

Druglikeness	
Lipinski	Yes; 0 violation
Ghose	No; 3 violations: MW>480, MR>130, #atoms>70
Veber	Yes
Egan	Yes
Muegge	Yes
Bioavailability Score	0.56

Medicinal Chemistry	
PAINS	0 alert
Brenk	1 alert: isolated_alkene
Leadlikeness	No; 2 violations: MW>350, XLOGP3>3.5
Synthetic accessibility	6.59

Figure S6. Prediction of tormentic acid (TA) properties based on the SwissADME database
